# Linking land use to the likely origins of third-generation cephalosporin-resistant Enterobacterales in freshwater

**DOI:** 10.1128/aem.00242-26

**Published:** 2026-06-26

**Authors:** Adrian L. Cookson, Rose M. Collis, Meg Devane, Jonathan C. Marshall, Marie Moinet, Amanda Gardner, Lynn Rogers, Sara Burgess, Patrick J. Biggs, Brent J. Gilpin

**Affiliations:** 1 New Zealand Institute for Bioeconomy Science, AgResearch Group, Hopkirk Research Institute, Massey University 6420, Palmerston North, New Zealand; 2 mEpiLab, School of Veterinary Sciences, Massey University 6420, Palmerston North, New Zealand; 3 New Zealand Institute for Public Health and Forensic Science, Christchurch, New Zealand; 4 School of Mathematical and Computational Sciences, Massey University 628177, Palmerston North, New Zealand; 5 School of Food Technology and Natural Sciences, Massey University 6420, Palmerston North, New Zealand; Centers for Disease Control and Prevention, Atlanta, Georgia, USA

**Keywords:** ESBL-*E. coli*, carbapenemase-resistant Enterobacterales, pAmpC, pAA, surface water, β-lactamase

## Abstract

Antimicrobial resistance is a major public health threat, as resistant infections are harder to treat and carry greater risks. Using a One Health framework, this survey of freshwater samples investigated the influence of local land use on the occurrence of waterborne third-generation cephalosporin-resistant Enterobacterales (3GC-E) mediated through extended-spectrum β-lactamases (ESBLs) or plasmid-mediated AmpC (pAmpC) genes. A cross-sectional survey of 339 freshwater samples from 49 New Zealand sites was undertaken, encompassing catchments with urban, agricultural (namely dairy; sheep and beef; and mixed dairy, sheep, and beef), avian-impact, and low-impact (exotic/native forest) dominant land uses. 3GC-E (*n* = 63), including *E. coli*, *Enterobacter*, *Citrobacter,* and *Klebsiella,* were isolated from freshwater samples (*n* = 35), where urban (32), dairy (1), avian (1), and sheep and beef (1) were identified as the dominant land use. 3GC-E genome assemblies (*n* = 43) from freshwater were compared with human ESBL-*E. coli* (*n* = 467) assemblies. Waterborne 3GC-*E. coli* were phylogenetically diverse, represented by seven phylotypes and 20 sequence types (ST131, ST38, ST68, and ST219) associated with human infections, suggesting contamination with urban wastewater. Two freshwater *E. coli* harbored the plasmid-associated carbapenemase-encoding *bla*_NDM-5_-*ble*_MBL_ gene cassette, which was absent from the human isolates. Two further freshwater *E. coli* and four human *E. coli* isolates carried pAA plasmids containing *agg* genes encoding for enteroaggregative *E. coli* adherence factors. By linking local land use at a national scale with the occurrence of freshwater 3GC-*E. coli*, we demonstrate the value of a One Health approach for understanding how human activities influence the environmental dissemination of antimicrobial resistance with implications for infectious disease risk.

**IMPORTANCE:**

Antimicrobial resistance (AMR) threatens public health by making infections harder and more expensive to treat. While AMR is often studied in clinical settings, environmental pathways that spread resistant bacteria remain less understood. Using a One Health approach, we investigated how land use influences the presence of third-generation cephalosporin-resistant Enterobacterales in freshwater across New Zealand. We found that resistant *Escherichia coli*, including strains carrying plasmid-associated virulence factors, were most frequently detected at sites impacted by urban land use and human fecal contamination. These findings show that human activities shape the environmental distribution of clinically relevant resistance and highlight freshwater as a potential exposure pathway. By linking national-scale land-use patterns with the occurrence of resistant bacteria in the environment, this study demonstrates how integrated environmental and public health surveillance can improve our understanding of AMR dissemination and inform strategies to reduce the spread of resistant pathogens.

## INTRODUCTION

Antimicrobial resistance (AMR) represents a global One Health emergency affecting humans, animals, and the environment, with the greatest burden falling on vulnerable populations (1, 2). Across these intertwined human–animal–environment compartments (3), inappropriate antibiotic use contributes to increased AMR and impacts the efficacy of antimicrobials vital to human and animal health (1, 4, 5). The World Health Organization Priority Pathogen list identifies key bacteria groups such as extended-spectrum beta-lactamase producing Enterobacterales (ESBL-E), and particularly *Escherichia coli* (ESBL-Ec) as gram-negative bacterial pathogens resistant to last-resort antibiotics requiring urgent attention to guide research, surveillance, and stewardship efforts (6, 7). The emergence of ESBL-E with the advent and increased use of critically important antibiotics (such as third- and fourth-generation cephalosporins [3GC/4GC]) highlights their suitability as an indicator organism and surveillance target for One Health case studies (8). Globally, these pathogens impose a substantial health and economic burden and limited treatment options, often requiring last-resort therapies, such as carbapenems or colistin (8). The most significant 3GC-antimicrobial resistant genes (ARGs) are the Class A *bla*_CTX-M_ ESBLs, typically carried on large mobilizable plasmids found across Enterobacterales and the *E. coli* phylogeny, particularly extra-intestinal pathogenic *E. coli* (ExPEC), conferring resistance to 3GCs such as cefotaxime (CTX) or cefpodoxime (CPD) (9). In contrast, Class C beta-lactamases include plasmid-associated AmpC (pAmpC) types, such as the *bla*_CMY_ genes conferring resistance to second-generation cephalosprins, including cefoxitin (FOX) (9).

Antimicrobial use in humans in New Zealand is very high compared with many other countries and has the fourth highest level of antibiotic prescription in the OECD (10, 11). Recent surveillance of 3GC-resistant Enterobacterales (3GC-E) from human clinical cases in New Zealand indicates increased prevalence, especially in more vulnerable populations (>65 years), with urine as the primary site of ESBL-E detection (12). Conversely, and unlike studies undertaken overseas where antibiotics have been permitted for prophylactic use in animal production (13, 14), the prevalence of 3GC-E from food animals and New Zealand pastoral farms is low (15–18) and reflects the relatively low veterinary antimicrobial use, especially those critically important antibiotics such as 3GC and 4GC, compared to overseas (18–20). The New Zealand Veterinary Association has set high aspirations to reduce the reliance on the use of antimicrobials for the maintenance of animal health and wellness in New Zealand by 2030 (21). Consequently, through prudent use of antimicrobials on New Zealand dairy farms, 3GC and 4GC are rarely used to treat mastitis or other diseases caused by gram-negative bacteria such as ESBL-E (19). Instead, the use of penicillins for dry cow therapy to reduce mastitis incidence and treat mastitic cattle during lactation is widespread as a routine animal welfare strategy in pasture-fed New Zealand dairy cattle (20). Extensive sampling of dairy farm environments, including cattle feces, farm effluent sources, bulk tank milk, and soil from recently grazed paddocks, also suggests low 3GC-E contamination levels (15, 17), but where sporadic veterinary use of 3GC, such as ceftiofur, occurs, the emergence of pAmpC *E. coli* has been observed (22).

Surveillance of freshwater samples, especially close to point source discharge of urban wastewater, can provide valuable information on the potential pathways of transmission of human 3GC-E to the receiving environment (23–25). Previous New Zealand studies, including whole genome sequencing (WGS) of AMR isolates, have identified the potential epidemiological links between ESBL-Ec, especially sequence type (ST) 131, isolated from freshwater sites impacted by wastewater discharge, and temporal ESBL-Ec isolated from human clinical isolates (25). Other studies have suggested links between urban land use and ESBL-Ec ST131 isolated from freshwater (26). Often, however, these prior studies have been limited in the extent of spatial sampling and addressing agricultural land use as a potential source of ESBL-Ec (26–28).

The aim of this study was to target 3GC-E from geographically widespread freshwater sample sites in New Zealand and examine the presence of ESBL and pAmpC genes and their possible association with a variety of dominant land uses. Importantly, sample sites were chosen that were specifically not influenced by human wastewater discharges to ensure that other dominant land uses, such as livestock production, and sites impacted by avian species, especially wildfowl, could be readily compared with low-impact land-use sites dominated by native or exotic forestry. The repeated cross-sectional study design incorporated repeated sampling of selected sites to address both site-specific contamination sources and the detection of transient or diffuse fecal sources. WGS was then used to link freshwater ESBL-Ec isolates with temporally aligned human clinical ESBL-Ec isolates, enabling high-resolution genome comparisons and ARG detection.

## MATERIALS AND METHODS

### Water sampling sites

The original water samples were collected as part of the 2020 Ministry for the Environment (MfE) Quantitative Microbial Risk Assessment (QMRA) Pilot (Phase 1) study (29) and from the 2022–2023 MfE Freshwater Pathogen (Phase 3) study (30). No samples were included from Phase 2 (31). For Phase 1, the samples were collected as part of a repeated cross-sectional pilot study from 16 sites (13 February to 19 March 2020) (Fig. S1). For Phase 3, the samples were taken to provide national coverage of different land uses from 41 sites as part of routine water quality monitoring programs by local government staff in late spring to early winter (October 2022 to July 2023) (Fig. S1). Sampling was scheduled to provide national coverage of different land uses, with site selection accounting for the logistics associated with long-term sampling across nine different Councils at 41 sites.

### Catchment delineation and land-use identification for freshwater sampling sites

The land use associated with sampling sites was determined as described previously (ArcGIS Pro, v.2.6.4) (30) to identify a hydrological sub-catchment for each sampling site. The catchment sizes ranged from approximately 3–8 km^2^, and land cover data (Table S1) for sites were sourced from the Land Cover Database version 5.0, Mainland, New Zealand (32) to assist in identifying the dominant land use for site categorization. Sites with a history of elevated concentrations of *E. coli* were located across New Zealand and reflected different dominant land uses and impacts as designated by Councils: Phase 1 (16 sites), dairy (*n* = 5); sheep and beef (*n* = 5); urban (*n* = 6) and Phase 3 (41 sites), dairy (*n* = 13); sheep and beef (*n* = 10); mixed sheep and beef and dairy (*n* = 2); urban (*n* = 8); avian (*n* = 4). Four further sites designated as “low impact” were included as sites with lower potential for fecal contamination (exotic/natural forest-dominated control sites). Sample sites were designated as “avian” using prior observational information, which noted large numbers of avian fecal contamination events caused by wildfowl flocks or bird roosts. Eight sites from Phase 1 (*n* = 4 dairy, *n* = 2 sheep and beef, and *n* = 2 urban) were included in the 41 sites sampled during Phase 3 (Fig. S1).

Water sampling was undertaken using aseptic methods across both baseflow and stormflow conditions using a repeated cross-sectional design, rather than being restricted to specific hydrological states, as described previously (30). Rainfall data for the preceding 24, 48, and 72 h were recorded for each site and visit for Phase 3 only and were included in the analysis. Samples were cooled and transported overnight for fecal indicator bacteria (FIB) analyses to the Institute of Public Health and Forensic Science (PHF Science, Christchurch, New Zealand, formerly ESR, the Institute of Environmental Research and Science).

### Enumeration and storage of fecal indicator bacteria

*E. coli* were analyzed in freshwater using Colilert Quanti-Tray/2000 assays (100 mL of a 1 in 10 dilution of water sample, incubated at 35°C ± 0.5°C for 24–28 h) (IDEXX Laboratories, Inc., Maine, US) (33). This method provides a detection range of <10 Most Probable Number (MPN) per 100 mL up to >24,200 MPN per 100 mL. Positive Colilert trays (*n* = 339; Phase 1: 42 water samples; Phase 3: 297 water samples) were transported (at 4°C) to the New Zealand Institute of Bioeconomy Sciences (AgResearch Group, Hopkirk Research Institute, Palmerston North) from PHF Science, Christchurch. Site identifier information was provided, but site locations were anonymous.

### Microbial source tracking (MST) markers

Freshwater samples (up to 1 L) were filtered (in duplicate) through a 0.45 µm mixed cellulose ester membrane filter (Millipore, France), and DNA was extracted using the PowerSoil Pro kit (Qiagen, Venlo, The Netherlands) as described previously (30). Quantitative PCR (qPCR) analyses were performed on a LightCycler 480 (Roche Diagnostics Ltd, California, US). The PCR targets are presented in Table 1, with PCR protocols and conditions outlined previously (34) and as described previously (30). Each qPCR run included negative and positive controls, and standard curves were generated from 10-fold serial dilutions ranging from 10^0^ to 10^6^ Gene Copies (GC) per 100 mL of the appropriate target. The qPCR assays were considered acceptable if their amplification efficiency was greater than 90% and their standard curve showed a coefficient of determination (*r*²) of at least 0.92.

**TABLE 1:** Target bacterial genes and methods for microbial source tracking qPCR

Fecal source host/target	Microbial target	Type of qPCR assay
General	Bacteroidales 16S rRNA (GenBac)	Probe-based (35)
Human	Duplex qPCR of *Bacteroides* HF183 and crAssphage CPQ_056	Probe-based (36)
	*Bifidobacterium adolescentis* (BiADO)	SYBR Green (37)
Ruminant	Bacteroidales 16S rRNA (BacR)	Probe-based (38)
Avian	GFD – Unclassified *Helicobacter* 16S rRNA gene	SYBR Green (39)

### Bacterial isolate identification and characterization

Bacterial growth from fluorescent blue Quanti-Tray wells was collected using aseptic methods and pooled for each individual tray. Briefly, growth was centrifuged (5,000 × *g*, 10 min), and the supernatant was discarded. The cell pellet was then resuspended in 1 mL EC broth and transferred to a 1.5 mL tube and centrifuged at 16,200 × *g* for 2 min. The supernatant was discarded, and the bacterial pellets were resuspended in 1 mL EC broth (Fort Richard, Auckland, New Zealand). The resuspended pellet was added to cryovials containing glycerol (final concentration 30% vol/vol) and stored at −80°C.

A loopful of frozen enrichment originating from each respective Quanti-Tray and stored at −80°C was inoculated onto Modified Charcoal Cefoperazone Deoxycholate agar (mCCDA) and incubated aerobically for 18–21 h at 35°C. Previous studies have demonstrated that mCCDA supports the selective growth of ESBL-Ec (40–42). Given its lower cost and the absence of biases associated with fermentation characteristics and subsequent colony color for the subsequent isolation of 3GC-E, it was selected as the primary selective medium to maximize 3GC-E recovery. At least two large white/cream colonies per enrichment were subcultured onto fresh mCCDA plates and then subsequently onto ECC (*E. coli*/Coliforms)-CHROMagar (Fort Richard, Auckland, New Zealand) and MacConkey agar (Fort Richard, Auckland, New Zealand) plates to assess beta-glucuronidase and beta-galactosidase activity. Individual isolates were stored in 1.5 mL EC broth containing 30% (wt/vol) glycerol at −80°C.

Bacterial identification was performed using the matrix-assisted laser desorption ionization-time of ﬂight (MALDI-TOF) mass spectrometry (Bruker, Billerica, CA, USA) (43) “on slide formic acid extraction” method (43) with the MBT Library BDAL 9.0 (Bruker Daltonics, Germany) library. Scores of ≥2 were indicative of species identification.

Primary evaluation for antimicrobial resistance was undertaken on Mueller-Hinton agar plates (Fort Richard, Auckland, New Zealand) using MASTDISCS containing cefotaxime (30 µg), cefoxitin (30 µg), cefpodoxime (10 µg), tetracycline (30 µg), streptomycin (10 µg) and ciprofloxacin (5 µg) (Fort Richard, Auckland, New Zealand). Antimicrobial susceptibility tests (ASTs) and confirmation of a 3GC-resistance phenotype were carried out using Kirby-Bauer disk diffusion assays following Clinical and Laboratory Standards Institute Guidelines (44).

The AmpC and ESBL beta-lactamase AMR phenotypes were confirmed for isolates resistant to either FOX and CTX and/or CPD using either a three-disc (D69C AmpC disc test, Mast Group Ltd., Liverpool, United Kingdom) or double-disc comparison assay (D62C cefotaxime and D64C ceftazidime ESBL disc tests, Mast Group Ltd., Liverpool, United Kingdom), respectively (45). The AmpC-producing *E. coli* NZRM4402 and the ESBL-producing *Klebsiella pneumoniae* NZRM3681 were used as positive controls in the AmpC and ESBL confirmatory disc assays, respectively, and the susceptible *E. coli* NZRM916 was used as a negative control. The carbapenemase resistant phenotype was confirmed using the MASTDISCS Combi Carba plus disc system for *Enterobacteriaceae* (D73C, Mast Group Ltd., Liverpool, United Kingdom).

### Whole genome sequencing and assembly

Genomic DNA was extracted from the 3GC-resistant Enterobacterales isolates grown in EC broth, using the Promega wizard genomic DNA purification kit as previously described (Madison, WI) (27). Sequencing was performed using both short-read and long-read technologies. For short-read sequencing, libraries were prepared using the Nextera XT DNA library preparation kit (Illumina Inc., San Diego, USA), and sequencing was performed using an Illumina HiSeq X with 2 × 125 bp paired-end reads (Novagene, Singapore).

Illumina raw sequence reads were processed using the Nullarbor (v. 2.0.20,191,013) pipeline (46), which included read trimming of adaptors (trimmomatic v. 0.39) (47), *de novo* genome assembly using SKESA (v.2.4.0) (48), bacterial classification using Kraken (v. 1.1.1) (49), and annotation using Prokka (v. 1.14.6) (50). 3GC-*E. coli* phylogenies were generated from core SNP alignments (with ESBL-Ec AGR6128 (SRR19180727) as the reference genome) generated with Snippy (v. 4.6.0) (51) using the Jukes-Cantor DNA evolution model. A phylogenetic tree was inferred by maximum likelihood using IQ-TREE (52), and the resultant tree was imported into the Interactive Tree of Life (v. 6.0) (53) software as a Newick file for subsequent annotation with experiment metadata for freshwater 3GC-*E. coli* (3GC-Ec). For phylogenetic analysis of the core SNP alignment of all 501 3GC-Ec isolates, the inferred IQ-TREE was visualized in GrapeTree (54).

ABRicate (v. 1.0.1) (55) was used for the mass screening of virulence using VFDB (Virulence Factor Database, dated 27 March 2021) (56), and antimicrobial resistance genes with ResFinder (v. 4.0) (57), with sequence identity and alignment coverage both set to default (>80%) settings. Point mutations conferring antibiotic resistance were identified using Resistance Gene Identifier/Comprehensive Antibiotic Resistance Database (RGI v.6.0.5, CARD v.4.0.1) (58). The sequence type of assembled 3GC-Ec contigs was performed using the Achtman MLST scheme (59) using PubMLST (60) (MLST, v. 2.19.0). SerotypeFinder (v.2.0) (61) was used to identify the *E. coli* serotype using assembled sequences data, while plasmid replicon sequences were identified using PlasmidFinder (v.2.1) (62). *E. coli* phylotypes were identified using the *in silico* ClermonTyping tool (63).

Long-read sequencing using Oxford Nanopore Technologies (ONT) was then carried out as previously described (15, 40). Libraries were prepared using the SQK-RBK114.24 kit and sequenced using R10.4.1 flow cells on an Oxford Nanopore Technologies MinION device using MinKNOW Core (5.7.2), followed by base-calling using Guppy (v.7.0.9) with the super-accurate R10.4.1. model. The ONT reads were demultiplexed using qcat (v.1.1.0), trimmed using porechop (v.0.2.4), and filtered using Filtlong (v.0.2.0), where 95% of the reads were kept with a minimum length of 1000 bp and a target number of bases of 500 Mb (~100× depth). Genome assemblies using long-read data only was carried out using Flye (v.2.9-b1768) (64) with default settings.

Core genome multi-locus sequence typing (cgMLST) was performed using ChewBBACA (v.3.4.1) (65) with the resultant cgMLST allelic profile data visualized in GrapeTree (54) with the MSTree v2 algorithm to construct a minimum spanning tree based on pairwise allelic distances. Plasmid sequences generated from long-read assemblies were visualized with BRIG –BLAST Ring Image Generator (66).

### Comparison with existing human ESBL-Ec isolates

In addition to the 3GC-E isolated in this study, *de novo* genome assemblies were generated in parallel using the same Nullarbor pipeline on WGS short-read data from a further 467 human ESBL-Ec clinical isolates obtained in two semi-contemporaneous studies (12, 25), for comparative purposes. The “ESR” data set consists of short-read (Illumina) data of 160 non-duplicate ESBL-Ec isolated from clinical specimens isolated between 18 August and 31 August 2019, inclusive (GenBank BioProject PRJNA1077717). The “MidCentral” data set consists of short-read (Illumina) data of 307 ESBL-Ec from urine, blood, or wound swab cultures and were isolated by the MedLab Central Laboratory (Palmerston North, Aotearoa New Zealand; samples processed from the Manawatū, Hawke’s Bay, Gisborne, and Whanganui regions), from August 2019 to March 2020, and June 2020 to January 2021 (GenBank BioProject PRJNA1032159).

### Data visualization and statistical analysis

Data visualization and statistical analysis were conducted in R (v.4.3.0) (67) using a range of tools, including ggpubr (v.0.60.0) (68) and ggplot2 (v.3.5.2) (69). Heatmaps were generated with ComplexHeatmap (v.2.18.0) (70) with dendrograms based on Euclidean distances between the prevalence profiles of the QMRA, ESR, and MidCentral data sets across all ARGs, clustered with complete linkage. igraph (v.2.1.14) was used for network analysis and visualization of ARGs identified from WGS data. Generalized linear mixed models (GLMMs; binomial family) were used to assess the associations between the presence of 3GC-E and land use, with log-transformed *E. coli* concentrations (log_10_MPN) included as a sample-level covariate. Associations between 3GC positivity and rainfall in the preceding 24, 48, and 72 h were also assessed, with rainfall log-transformed (log_10_[rainfall + 1]) to account for zero values. In all models, site was included as a random intercept to account for non-independence among samples. For the association of 3GC-E with land use, categories were aggregated into “urban” and “non-urban” to provide a clear analytical contrast and ensure adequate sample size within groups. The “non-urban” category includes sites with differing land uses and therefore represents a simplified classification. Models were fitted using the lme4 package (71).

## RESULTS

In total, 339 water samples were obtained during the two separate studies: 42 samples from 16 sites in Phase 1 and 297 samples from 41 sites in Phase 3 (Fig. S1). For Phase 1, 11 water samples were from sites where sheep and beef farming were the dominant land use, 14 from dairy, and 17 from urban. During Phase 3, water samples were from sites with the following dominant land use: 80 urban, 32 avian, 69 dairy, 25 low impact, 21 mixed sheep, beef, and dairy, and 70 sheep and beef. There was no significant difference between *E. coli* counts from urban (666.5 geometric mean MPN per 100 mL) and dairy (533.3 geometric mean MPN per 100 mL) sample sites (Fig. S2), but concentrations from other land uses were significantly lower (*P* < 0.001) than urban, with low-impact samples having the lowest concentration (73.1 geometric mean MPN per 100 mL, Fig. S2).

**Fig 1:**
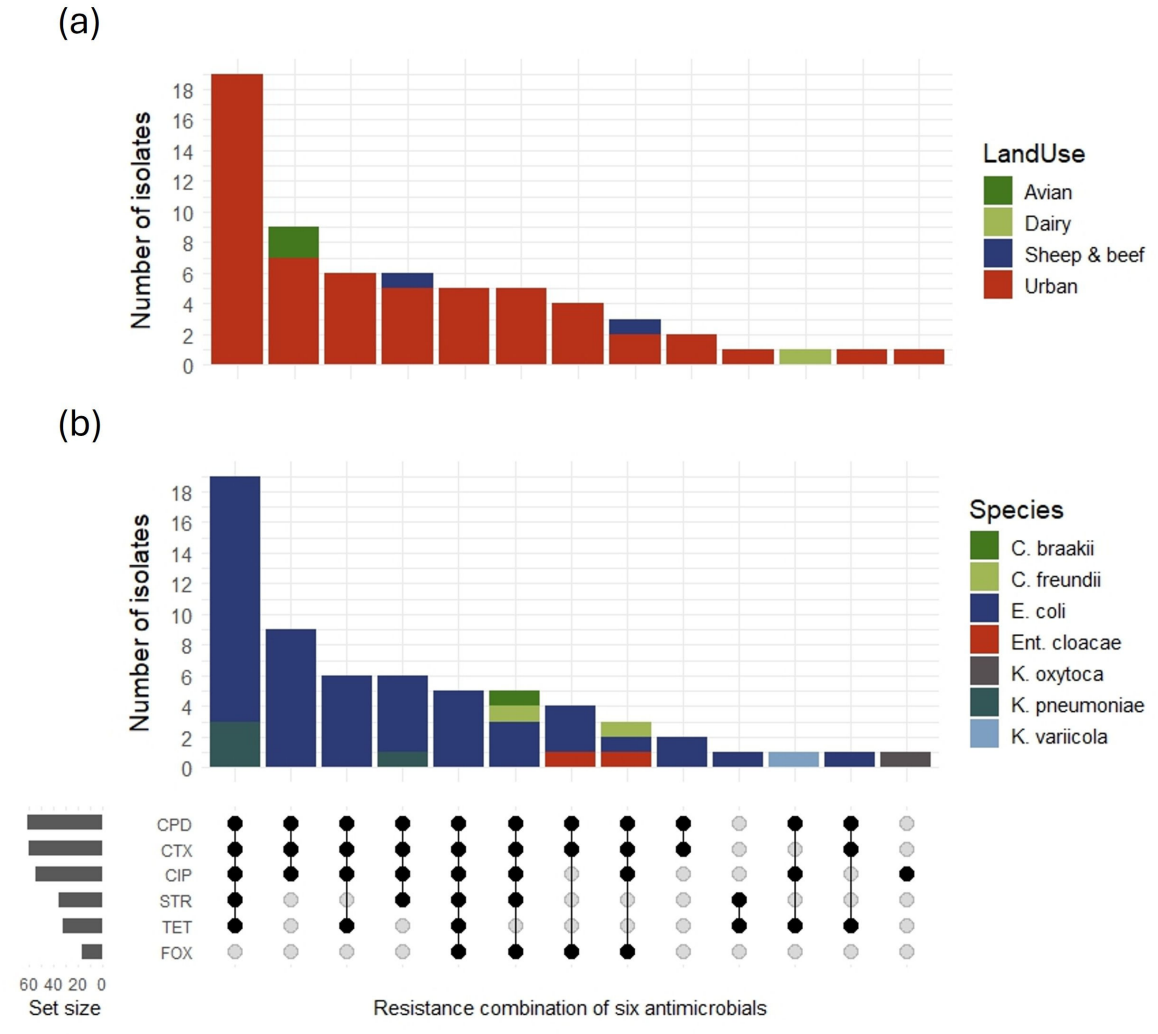
Antimicrobial resistance phenotypes of ESBL-producing Enterobacterales against six antibiotics from (**a**) sample sites associated with different dominant land uses and (**b**) separated according to microbial species identified using MALDI-TOF mass spectroscopy.

**Fig 2:**
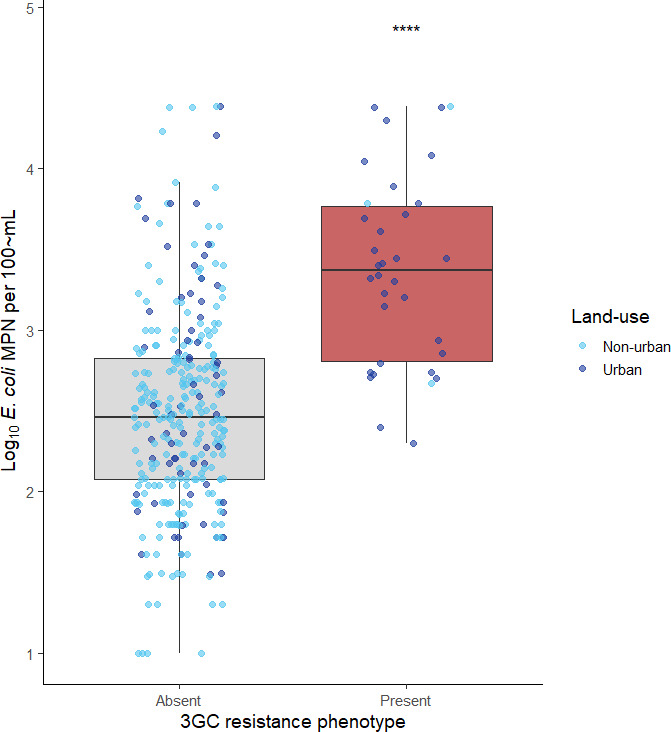
*E. coli* Log_10_ MPN per 100 mL concentrations across sites. Box and whisker plot of log_10_-transformed freshwater *E. coli* (MPN per 100 mL) counts (*N* = 339) separated according to whether Enterobacterales isolates resistant to the third-generation cephalosporins cefpodoxime and/or cefotaxime (3GC-E) were present from each of the samples across different dominant land uses. Points represent individual measurements from non-urban and urban land use. The boxes show median values and span lower to upper quartiles, the whiskers show the highest and lowest values within 1.5 times the interquartile range, and dots beyond the whiskers show potential outliers. Higher Log_10_MPN values were observed in samples with a 3GC-E-positive phenotype compared to those samples from which no 3GC-E isolates were obtained (Fisher’s exact test, *P* < 0.001).

Although most putative 3GC-E isolates (92.1%, 58/63) were isolated from 33.0% (32/97) urban freshwater samples, two isolates (3.2%, 2/63) were isolated from an avian sample (3.1%, 1/32), two isolates (3.2%, 2/63) from a sheep and beef sample (1.2%, 1/81), and a single isolate (1.6%, 1/63) from a dairy freshwater sample (1.2%, 1/83) (Fig. 1a; Table S2). Across urban sites, 3GC-E was found at 80% (8/10) of locations, with a particularly high prevalence at urban site U3, where 73.3% (11/15) of samples collected between February 2020 and June 2023 were positive (Table S2). However, no 3GC-E were isolated from other urban sites, including U2 (sampled on 9 occasions), U7 (9 occasions), or U8 (12 occasions) (Table S2). Due to the scarcity of isolates with a 3GC-resistance phenotype from dairy, avian, low-impact, sheep and beef, and mixed sheep, beef, and dairy land uses, these land uses were combined into a non-urban reference group. Both land use and *E. coli* concentrations had a statistically supported association with 3GC-E detection when included as co-variates; after adjusting for log_10_-transformed MPN values, samples from urban sites had an observed association of 3GC-E detection (OR 72.8, CI: 7.9–666.5, *P* = 0.0001). Increased *E. coli* concentrations were also associated with higher odds of detection (log_10_MPN; OR 11.1, CI: 3.4–35.4, *P* < 0.0001) (Fig. 2). The association between total rainfall and the 3GC phenotype for Phase 3 water samples was consistent across time periods, with similar effect sizes observed for rainfall in the preceding 24, 48, and 72 h (24 h: OR 2.08, 95% CI: 1.21–3.58, *P* = 0.008; 48 h: OR 2.02, 95% CI: 1.24–3.31, *P* = 0.005; 72 h: OR 2.13, 95% CI: 1.31–3.48, *P* = 0.002).

Human was a dominant or co-dominant MST signal in 30 of 35 (85.7%) water samples from which putative 3GC-E were isolated (Table S2). For three samples where *Citrobacter* (*n* = 2) or *Klebsiella* (*n* = 1) were isolated, the dominant urban land use did not match the dominant MST marker (ruminant or avian). For the final two non-human MST samples, there was concordance between MST data and dominant land use (e.g., avian MST and avian land use, ruminant MST, and dairy land use).

Most of the mCCDA-positive isolates (82.5%, 52/63) were identified as *E. coli* using MALDI-TOF analysis, with the remaining 11 isolates identified as *Citrobacter* spp. (*n* = 3), *Enterobacter cloacae* (*n* = 2), and *Klebsiella* spp. (*n* = 6) (Fig. 1b; Table S2). However, only 30% (19/63) isolates obtained from mCCDA were both β-galactosidase-positive (purple colonies on MacConkey agar) and β-glucuronidase-positive (blue colonies on ECC-CHROMagar) (Table S2). Other fermentation profiles included 33% (21/63) isolates that were β-galactosidase-negative (gray colonies on MacConkey agar) and 25 isolates (39.7%), which were β-glucuronidase-negative (pink colonies on ECC-CHROMagar), including two putative 3GC-E isolates from one sample that were both β-galactosidase- and β-glucuronidase-negative (Table S2).

AMR phenotypes were examined using ASTs against cefpodoxime (3.2%, 2/63 susceptible), cefotaxime (4.8%, 3/63 susceptible), cefoxitin (71.4%, 45/63 susceptible), tetracycline (47.6%, 30/63 susceptible), streptomycin (42.9%, 27/63 susceptible), and ciprofloxacin (12.2%, 8/63 susceptible, Table S3). Most isolates (65.1%, 41/63) were multi-drug-resistant (MDR), that is, resistant to three or more antibiotic classes (β-lactams, aminoglycosides, tetracyclines, or fluoroquinolones) (Fig. 1b; Table S3). Although isolated from mCCDA that contains cefoperazone, a 3GC, two isolates (*K. oxytoca* AGR7385 and *E. coli* AGR7367) were susceptible to CPD and CTX and were therefore not deemed as 3GC-E, while *K. variicola* AGR7372 was susceptible to CTX but resistant to CPD; hence, it did not fit the ESBL phenotype definition (Table S3).

Overall, the ESBL (Class A beta-lactamase) and AmpC (Class C beta-lactamase) enzyme AST phenotypes were confirmed for all isolates using the double-disc ESBL and three-disc AmpC tests, respectively, except for AGR7387 (FOX resistant), where the AmpC phenotype could not be confirmed using the three-disc AmpC test (Table S3).

### Genomic epidemiology of 3GC-resistant Enterobacterales

A total of 63 3GC-resistant isolates were isolated from 35 water samples. Where possible, at least two separate colonies from mCCDA plates were sub-cultured and stored for further analysis. According to AST and fermentation characteristics, 20 isolates were considered duplicates and were not included in the panel of 43 freshwater isolates (34 *E. coli*, 4 *K. pneumoniae*, 3 *Citrobacter* spp., 1 *K. oxytoca*, and 1 *Enterobacter* sp.) that underwent WGS (Table S4). Freshwater isolates for WGS were selected to ensure representative isolates with unique AMR phenotypes using AST data were included. Overall, the culture (MALDI-TOF) and WGS-based methods were concordant for isolate identification except for AGR7372, which was identified as *K. variicola* using MALDI-TOF MS and *K. pneumoniae* using Kraken (Table S5). The most common freshwater ESBL-type was *bla*_CTX-M_, which was identified in 86.0% (37/43) of ESBL-E isolates. The predominant ESBL-coding gene was *bla*_CTX-M-15_ (*n* = 21), followed by *bla*_CTX-M-27_ (*n* = 6) and *bla*_CTX-M-14_ (*n* = 5), with clear linkages between *bla*_CTX-M-15_ and *bla*_CTX-M-27_ with other ARGs as displayed through network analysis (Fig. S3). In total, 81.4% (35/43) isolates that underwent WGS had an MDR genotype.

**Fig 3:**
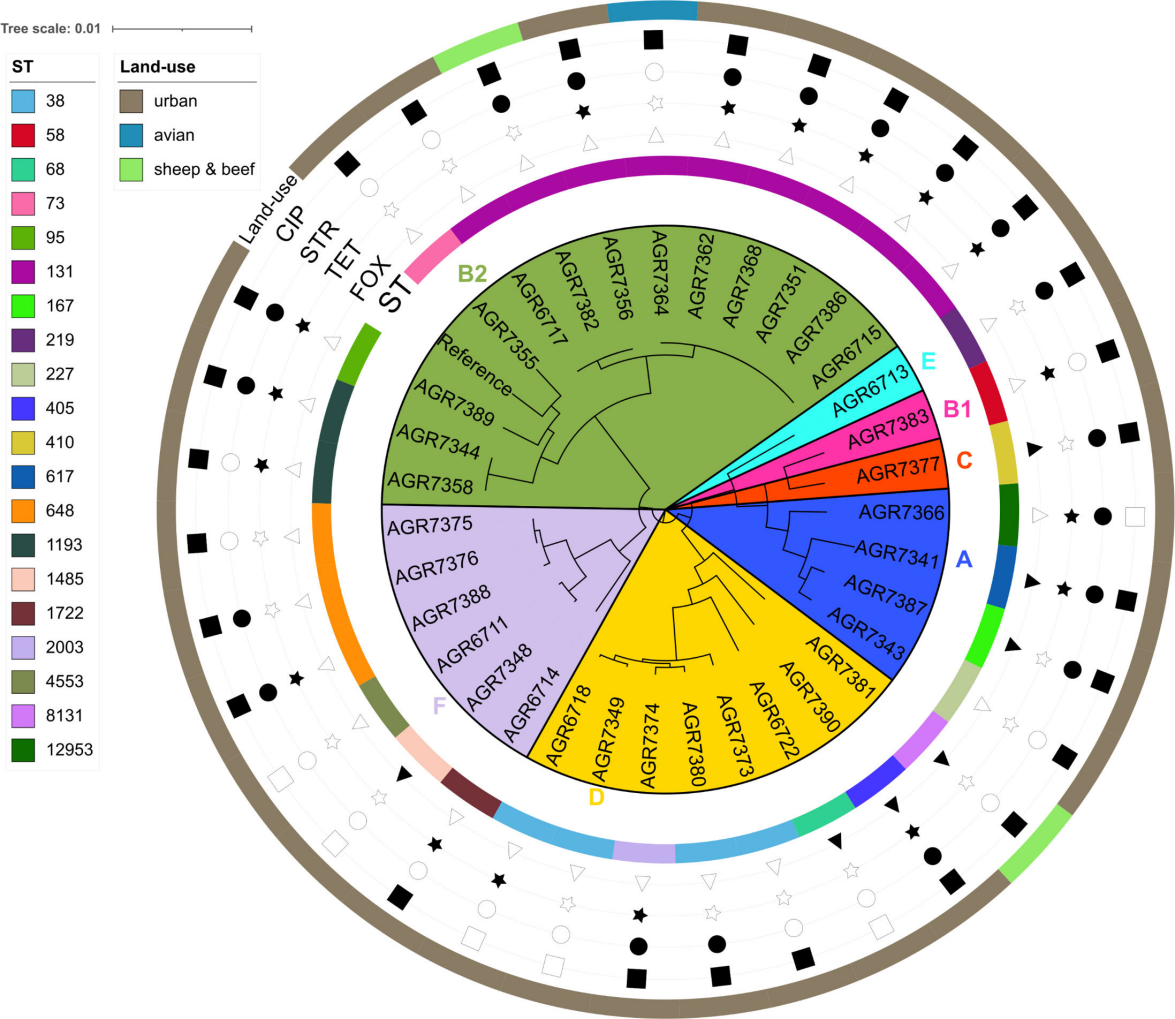
Maximum-likelihood tree, visualized using the Interactive Tree of Life tool and rooted at the mid-point of the core genome single-nucleotide polymorphism phylogeny of third-generation cephalosporin resistant *E. coli* (3GC-*E. coli*) isolates (*n* = 34) isolated from freshwater. The genetic diversity of 3GC-*E. coli* were examined by generating core SNP alignments using Snippy (v. 4.6.0) with ESBL-*E. coli* AGR6128 (SRR19180727) as the reference genome. The Jukes-Cantor substitution model was used to undertake evolutionary analysis of the core SNP alignment, and a phylogenetic tree was inferred by maximum likelihood using IQ-Tree with the resultant tree imported into the Interactive Tree of Life tool as a Newick file. Clustering broadly separated 3GC-*E. coli* isolates into discrete phylotypes (innermost colored circle). Moving outward, the next ring represents multi locus sequence type (ST), presence/absence of the cefoxitin (FOX) resistance phenotype, tetracycline (TET) resistance phenotype, streptomycin (STR) resistance phenotype, and ciprofloxacin (CIP) resistance phenotype. The outermost ring signifies the dominant land use of each freshwater sample site.

Single-nucleotide polymorphism (SNP) analysis of *E. coli* (*n* = 34) using 215,106 SNPs (4.2% of average genome size, 5.124 Mb) separated the isolates into phylogenies representing seven distinct phylotypes (Fig. 3) including phylotype A (*n* = 4), B1 (*n* = 1), B2 (*n* = 13), C (*n* = 1), D (*n* = 8), E (*n* = 1), and F (*n* = 6). Using PubMLST, 20 different sequence types (ST) were identified from *E. coli* data, with 16 ST being represented by singleton isolates only and ST131 (*n* = 9) being the most abundant (Fig. 3), separated into two distinct groups, Clade B (O16:H5), and Clade C (O25:H4) (72) (Table S5), according to serotype.

### Phenotype and genotype concordance

The relationship between AMR genotype and phenotype was examined in more detail, targeting ARGs associated with the resistance phenotypes against CPD, CTX, FOX, STR, TET, and CIP (Table S5). A non-susceptible phenotype (proportion of test isolates exhibiting intermediate and resistant phenotypes combined) to CPD and CTX was associated with carriage of beta-lactamase-encoding *bla*_CTX-M_ genes. Four 3GC-Ec isolates with a FOX resistance phenotype possessed pAmpC *bla*_CMY_ genes (AGR6722, *bla*_CMY-2_; AGR7348, *bla*_CMY-2_; and AGR7387, *bla*_CMY-145_), and *bla*_DHA-1_ from AGR7381 was chromosomally encoded. Although AGR7390 displayed a FOX-resistance phenotype, it was negative in the three-disc AmpC test, and no AmpC genes were identified from short- and long-read WGS data. AGR7341 and AGR7377 were resistant to CTX and CPD and exhibited a FOX-resistant phenotype but were not inhibited by clavulanic acid (required for positive ESBL confirmation, due to the presence of *bla*_NDM-5_, which is not inhibited by clavulanic acid) (Table S5).

Most (94.4%, 17 of 18) 3GC-E isolates with tetracycline ARGs (*tet*A, *tet*B, *tet*D, and *tet*X) displayed a tetracycline-resistant phenotype except for the *tet*A-positive isolate AGR7377, which was tetracycline susceptible during AST analysis (Table S3). Linking genotype and phenotype for the ciprofloxacin resistant isolates was more complex due to the combination of plasmid-mediated quinolone resistance (PMQR) genes (*qnr*B, *qnr*S variants) and also SNPs in the quinolone resistance determining region (QRDR: *gyr*A, *par*C). Increased AST zone sizes against ciprofloxacin were in isolates with a *gyr*A (D87N, S83L), *par*C (S80I) genotype (Fig. S4). Larger AST zones, still indicative of a resistance phenotype, were in isolates with a *gyr*A (S83L) genotype and/or *qnr*B/*qnr*S variants (Fig. S4). ST131 isolates were able to be separated into two separate groups according to ciprofloxacin AST results and ARG profiles that matched Clade B (larger AST inhibition zones and a *gyr*A [S83L] genotype and/or *qnr*B/*qnr*S variants ARG profile) and Clade C (nominal AST inhibition zone and a *gyr*A (D87N, S83L), *par*C (S80I) genotype) concomitant with separate serotypes (Clade B, O16:H5; Clade C, O25:H4) and phylogenies (Table S5).

**Fig 4:**
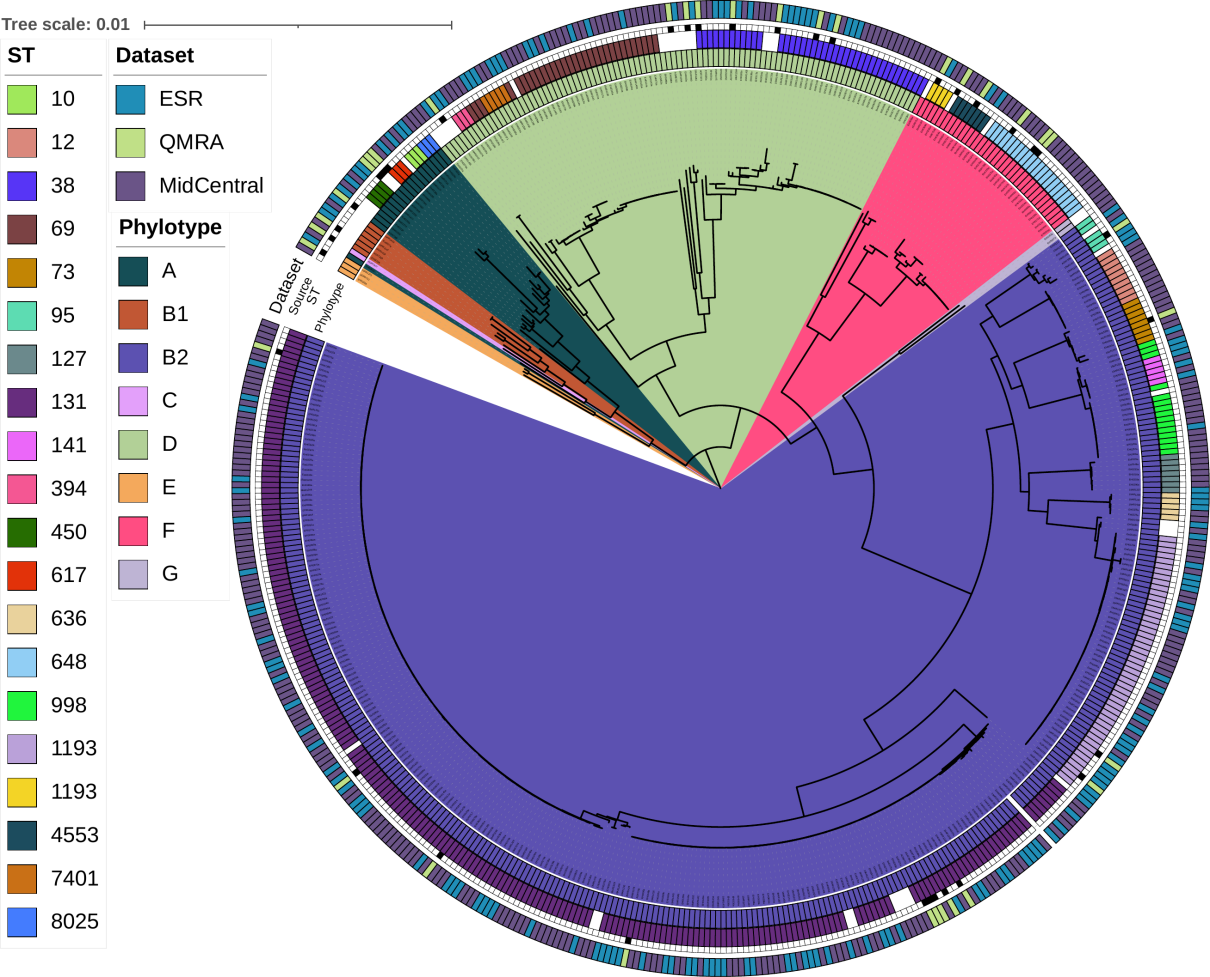
Maximum-likelihood tree of the core genome single-nucleotide polymorphism phylogeny of third-generation cephalosporin-resistant *E. coli* (3GC-*E. coli*) isolates (*n* = 501) isolated from freshwater or human clinical cases. The genetic diversity of 3GC-*E. coli* were examined by generating core SNP alignments using Snippy (v. 4.6.0) with ESBL-*E. coli* AGR6128 (SRR19180727) as the reference genome. The Jukes-Cantor substitution model was used to undertake evolutionary analysis of the core SNP alignment, and a phylogenetic tree was inferred by maximum likelihood using IQ-tree with the resultant tree rooted at the midpoint and imported into the Interactive Tree of Life tool as a Newick file. Branch lengths correspond to the number of core SNP differences between isolates, with the scale bar indicating the proportion of nucleotide substitutions per site. Closely related clusters suggest clonal relationships with 3GC-*E. coli* isolates separated into discrete phylotypes (innermost colored sectors). Moving outward, the next ring represents multi-locus sequence type (ST) of the 20 most abundant STs, sample source (freshwater black squares), and WGS data set.

Illumina short-read data from this study were processed in parallel with short-read data from two recent New Zealand data sets comprising ESBL-Ec from clinical sources (*n* = 307 and *n* = 160) to explore the links between ESBL-Ec from humans and surface water samples. Together with the 34 3GC-Ec from this study, a combined data set of 501 isolates was generated. Core SNP alignments (Fig. 4) and cgMLST (Fig. S5) were used to compare phylogenies between the three data sets, with both demonstrating dispersal of freshwater isolates throughout the phylogeny. SNP analysis across the 501 3GC-Ec genomes using 180,306 SNPs (3.51% of average genome size, 5.14Mb) separated isolates into phylogenies representing distinct phylotypes including B2, D, and F, which were the most abundant, and A, B1, C, E, and G, which were less prevalent (Fig. 4). The cgMLST minimum spanning tree was constructed from 3064 core loci at 95% similarity level and separated clusters according to ST but with less concordance to separate *E. coli* phylotypes (Fig. S5).

Across the 501 3GC-Ec genomes, at least 62 different STs were identified (Table S4). Compared to the 34 isolates from this study, which encompassed 20 STs, including ST131 (9/34, 26.5%), the ESR data set included 33 STs, with ST131 being the dominant ST (71 of 160, 44.4%), while the MidCentral data set contained 35 STs, with ST131 again the most dominant ST (138/307, 44.9%) (Table S4). The predominant STs across all 501 3GC-Ec isolates were ST131 (218/501, 43.5%), ST1193 (49/501, 9.8%), ST38 (40/501, 8.0%), ST69 (31/501, 6.2%), ST648 (22/501, 4.4%), and ST998 (15/501, 3.0%), with these six STs representing 74.9% of all isolates. Including ST131, there were eight STs (ST38, ST73, ST95, ST617, ST1193, ST1722, and ST4553) shared among the three datasets (Table S4). There were eight freshwater-only STs (ST68, ST167, ST227, ST405, ST410, ST1485, ST2003, and ST12953), 10 ESR human-only STs, and 14 MidCentral human-only STs.

**Fig 5:**
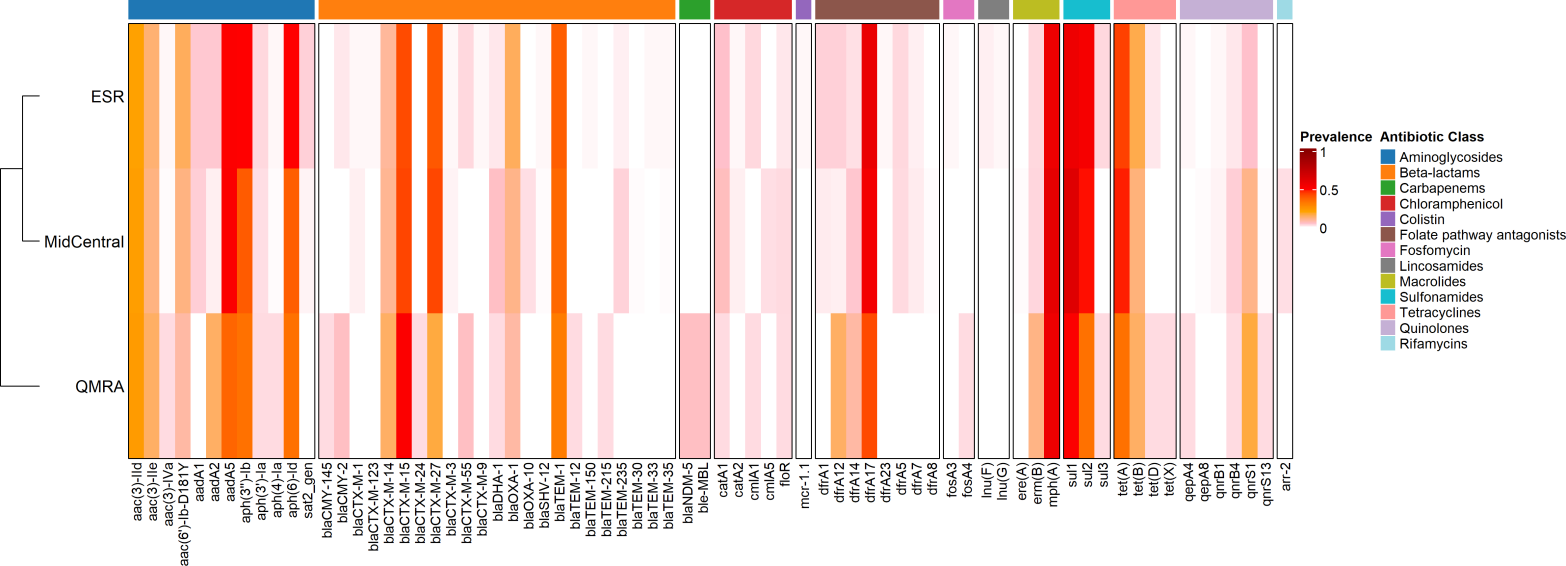
Prevalence of ARGs across *E. coli* isolate collections from QMRA (*N* = 34), ESR (*N* = 160), and MidCentral (*n* = 307) data sets. Heatmap showing the percentage prevalence of individual ARGs and ARG families detected among *E. coli* isolates from the QMRA, ESR, and MidCentral data sets. Each row represents a data set, and each column corresponds to a specific ARG or ARG family. Color intensity indicates the prevalence of each gene within a data set, with darker shades representing higher prevalence. The heatmap illustrates variation in ARG profiles between data sets, including consistently detected β-lactamase (*bla*_CTX-M-15_), aminoglycoside (*aad*A5, *aph*(3’’)-Ib), folate pathway antagonist (*dfr*A17), sulfonamide (*sul*1), and tetracycline (*tet*(A)) resistance determinants.

In total, 79 different ARGs were identified, with 35 (44.3%) shared among the three datasets, and 8 or 9 (10 or 11%) unique to each data set (Fig. 5; Table S8). The prevalence of the most common ARGs such as *aad*A5, *aph*(3'')−Ib, *bla*_CTX−M−15_, *bla*_CTX−M−27_, *dfr*A17, *sul*1, *sul*2, and *tet*A was broadly similar in isolates across the three datasets (Fig. 5). The *bla*_CTX−M−15_ gene was the most common ESBL gene, and although the *bla*_CTX−M−27_ was the second most common ESBL-gene, it was much less prevalent in the freshwater ESBL-Ec isolates compared to the human clinical isolates (Fig. 5). Other less prevalent ARGs such as *bla*_NDM-5_, *mcr*1.1, *fos*A4 and *arr*2 were typically confined to a single data set only (Fig. 5; Table S7).

Thirty-three 3GC-Ec also underwent sequencing using ONT with long-read *de novo* assemblies. Most genome assemblies, including plasmids, consisted of ≤5 contigs (81.8%, 27/33) including two isolates (AGR6718 and AGR7349) with assemblies that consisted of a single closed chromosome (Table S6). High-resolution assemblies of long-read data permitted the identification of whether ARGs were plasmid or chromosome-associated. *bla*_CTX-M_ genes were plasmid encoded in 11 ESBL-Ec and located chromosomally in 19 ESBL-Ec, including two ST131 Clade B isolates AGR7351 and AGR7362 with two chromosomal copies of *bla*_CTX-M-27_, and AGR6717 with a single plasmid-associated *bla*_CTX-M-14_, and a chromosomal *bla*_CTX-M-15_ copy (Tables S5 and S6). In addition to pAmpC-encoded genes *bla*_CMY-2_ and *bla*_CMY-145_, *bla*_DHA-1_ from AGR7381 was chromosomally encoded (Table S5). Also, for one ESBL-Ec isolate (AGR7389), the *tet*A and *tet*X genes were located on the same plasmid (Table S6). Plasmids were diverse and ranged in size from small colicin-producing plasmids, including Col(BS512) (2.0 kb), to large plasmids of up to 223 kb (Table S6). Large Col156/IncFIB/IncFII replicon type plasmids (146.7–149.5 kb) associated with ESBL-Ec ST131 Clade B isolates were nearly identical (Fig. S6; Table S6) and displayed an MDR genotype [*aac* (3)-Ild, *aph* (6)-Id, *aph*(3'')-Ib, *aad*A5, *bla*_TEM-1B_, *mph*(A), *sul*1, *sul*2, *tet*(A), *dfr*A17]. In contrast, the long-read assembly of the MDR AGR7380 (ST38) isolate indicated the presence of a large (136,474 bp) Col156/IncFIB/IncFII replicon type plasmid, which was devoid of ARGs. However, genes involved in aminoglycoside [*aac* (3)-IId*, aad*A2*, aph*(3'')-Ib*, aph* (6)-Id], beta-lactamase (*bla*_CTX-M-15_), trimethoprim (*dfr*A12), macrolide (*mph*A), and sulfonamide (*sul*1, *sul*2) resistance were located on the chromosome. Another large plasmid, pAGR7389a (202,481 bp), contained virulence genes involved in aerobactin-mediated iron acquisition (e.g., *iro*N, *iuc*A, *iut*A, and *sit*A) in addition to genes mediating in aminoglycoside, chloramphenicol, trimethoprim, and sulfonamide resistance (Fig. S7). Small colicin-producing plasmids of between 2.0 and 12.7 kb were identified from 7 ESBL-Ec that underwent long-read sequencing and included plasmid replicon types Col(BS512) (*n* = 3), ColpVC (*n* = 2), Col156 (*n* = 2), and Col440II (*n* = 1), with one isolate, AGR7375, having Col156 and ColpVC (Table S6).

**Fig 6:**
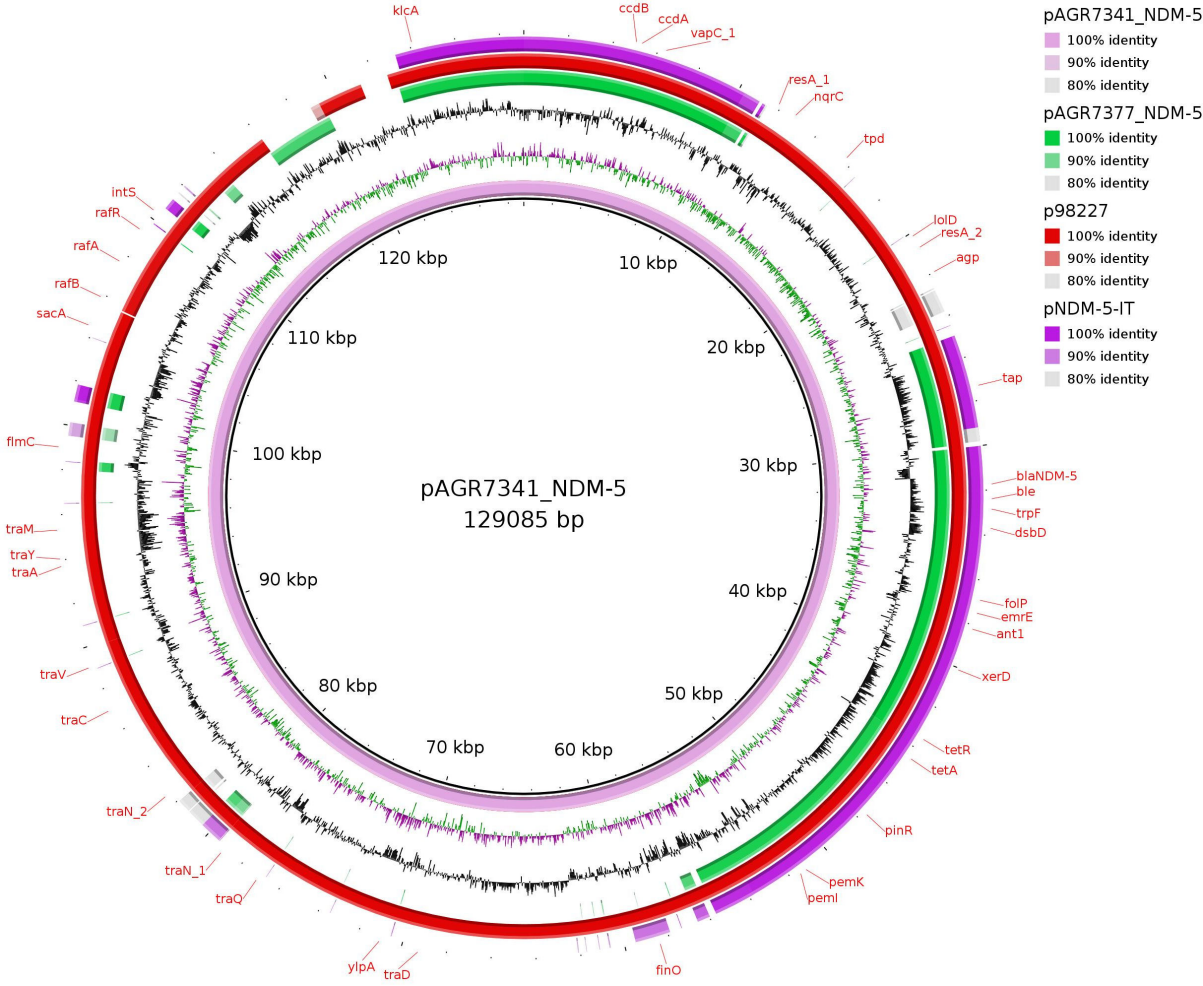
Comparative plasmid analysis of four *bla*_NDM-5_-containing plasmids using BRIG (BLAST Ring Image Generator). The circular map illustrates the structural comparison of four plasmids harboring the *bla*_NDM-5_ gene. The innermost ring represents the reference plasmid pAGR7341a (IncFIA, IncFII) from ESBL-E. coli AGR7341, while concentric outer rings depict plasmids pAGR7377a (IncFIA, IncFIB, and IncFII) from ESBL-E. coli AGR7377, p98227 (GenBank accession CP173469), and pNDM-5-IT (GenBank accession MG649062) aligned by BLASTn similarity. Color intensity of the rings reflects sequence identity, with darker shading indicating higher similarity. The map highlights regions of conservation and divergence among the plasmids, showing shared *bla*_NDM-5_ loci and variable accessory regions, including additional resistance determinants.

Among the ESBL-Ec isolates that underwent long-read sequencing, two surface water isolates (AGR7341, ST617 and AGR7377, ST410), both from site U5 associated with urban land use, had genes associated with carbapenemase activity mediated by *bla*_NDM-5_ and confirmed carbapenemase-resistant phenotypes. In both isolates, the *bla*_NDM-5_ gene was located adjacent to *ble*_MBL_ and flanked by multiple insertion sequences (IS26, IS6100, and ISSsu9) and transposases (ISSba14 and TnAs1). The gene was carried on MDR plasmids of 129 kb and 98 kb, corresponding to IncFIA/IncFII and IncFIA/IncFIB/IncFII replicon types, respectively (Table S6). Despite sharing similar structural features, the two plasmids showed low overall sequence identity (Fig. 6). However, the *bla*_NDM-5_ plasmid from AGR7341 showed regions of increased overall sequence identity to a *bla*_NDM-5_ containing plasmid (p98227, GenBank accession CP173469) from a human clinical *E. coli* isolated in Egypt, whereas the *bla*_NDM-5_ plasmid from AGR7377 was more similar to a *bla*_NDM-5_ containing plasmid (pNDM-5-IT, GenBank accession MG649062) from an *E. coli* isolated from a human clinical UTI case in Italy. No *bla*_NDM-5_ genes were identified from Illumina short-read data generated from the “ESR” and “MidCentral” New Zealand human clinical isolate collections included in this study.

**Fig 7:**
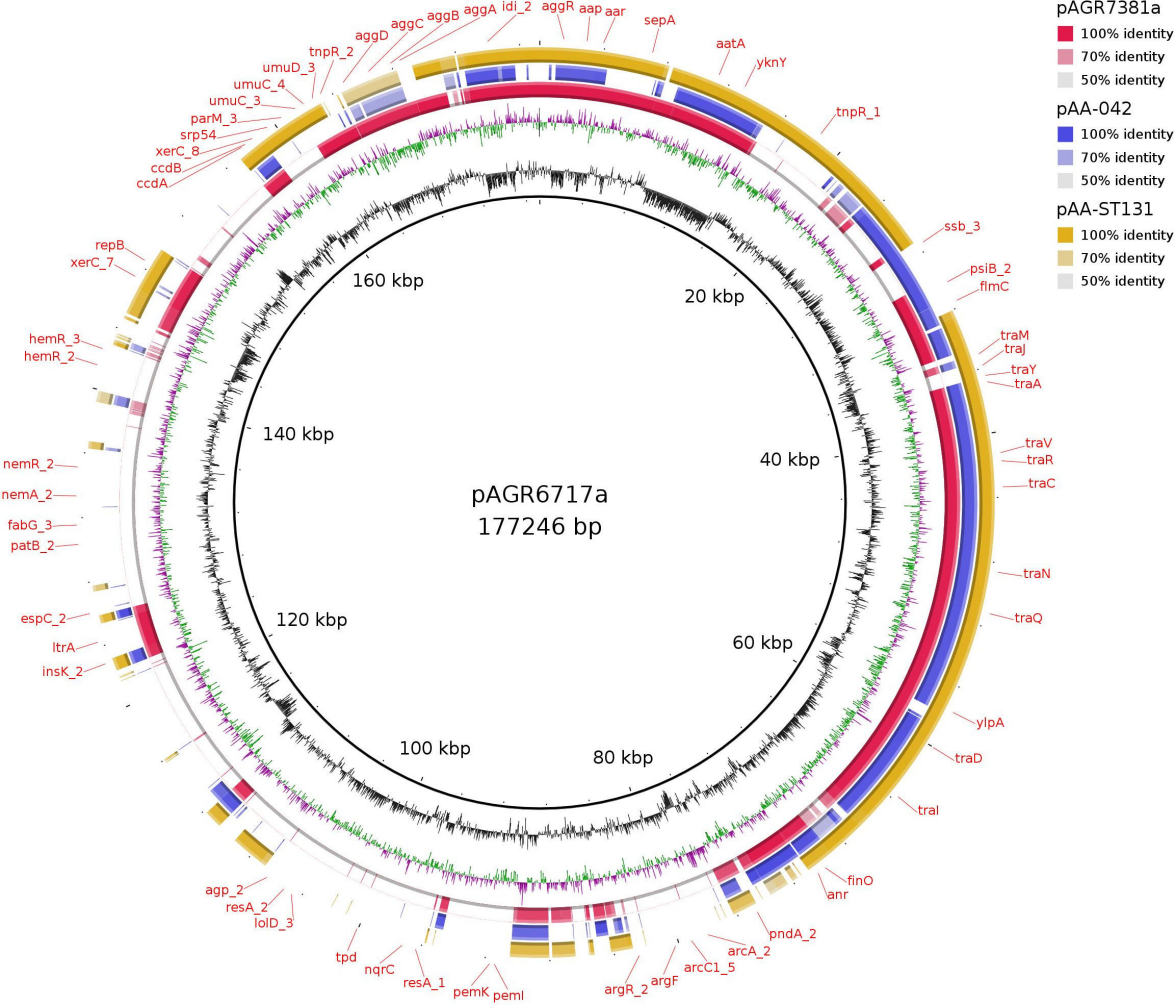
Comparative plasmid analysis of aggregative adherence plasmids using BRIG (BLAST Ring Image Generator). The circular map illustrates the structural comparison of four plasmids harboring the aggregative adherence genes. The innermost ring represents the reference plasmid pAGR6717a (IncFIB, IncFII) from ESBL-*E. coli* AGR6717, while concentric outer rings depict plasmids pAGR7381a (IncFIB, IncFII) from ESBL-*E. coli* AGR7381, pAA_042 (FN554767), and pAA_ST131 (KY706108) aligned by BLASTn similarity. Color intensity of the rings reflects sequence identity, with darker shading indicating higher similarity. The map highlights regions of conservation and divergence among the plasmids, showing shared *agg* loci and variable accessory regions.

Further analysis of the virulome from WGS data using VFDB also indicated the presence of the enteroaggregative *E. coli* virulence plasmid (pAA)-associated *agg* genes, encoding factors for enteroaggregative adherence in two isolates (AGR6717, ST131 and AGR7381, ST8131) (Tables S6 and S8). AGR6717 was isolated from a freshwater sample obtained from site P1 associated with urban land use, whereas AGR7381 was isolated from site Sh4 associated with sheep and beef production land use. While both plasmids contained the *aap, aar, aat*A*, agg*A*, agg*B*, agg*C*, agg*D*, agg*R, and *anr* genes, plasmid sizes differed by over 40 kb (pAGR6717, 177,246 bp and pAGR7381, 134,203 bp) (Table S6) with limited identity to each other and also with other *E. coli* pAA plasmids: pAA_042 (FN554767) and pAA-ST131 (KY706108) (Fig. 7). *agg* genes were also identified in WGS data from four human isolates, including an ST8131 from the ESR data set that had 21 SNPs different from AGR7381 (ST8381), two ST394 from the MidCentral data set which differed from each other by 8 SNPs, and an ST127 from the MidCentral data set.

Although the 501 isolates represented considerable diversity at the ST level, at the SNP level, there were some indications of phylogenetic similarity (Table S9). For example, three clinical isolates (one from MidCentral and two from ESR collection) differed from freshwater ESBL-Ec AGR6711 (*bla*_CTX-M-15_) by < 10 SNPs. Similarly, two isolates from the ESR collection differed from the MDR ESBL-Ec freshwater isolate AGR6715 by < 10 SNPs (Table S9).

Two plasmid-associated *bla*_CTX-M_ genes were identified from non-*E*. *coli* ESBL-E including *bla*_CTX-M-3_ (*Citrobacter* sp. AGR7353, AGR7360, and AGR7361), and *bla*_CTX-M-15_ (*K. pneumoniae* AGR7346 and AGR7370) (Table S6). Other beta-lactamase genes included *bla*_LEN-17_ (*K. pneumoniae* AGR7372) and a Class C beta-lactamase *bla*_LAP-2_ (*K. oxytoca* AGR7385), which were negative in the ESBL and AmpC confirmatory tests, respectively (Table S3). The *bla*_LEN-17_-positive isolate was negative in the ESBL double-disc confirmatory test, reflecting the narrow-spectrum activity of this class A β-lactamase (Table S3). Similarly, the *bla*_LAP-2_-positive isolate remained susceptible to FOX and was negative in the AmpC three-disc test, consistent with weak or low-level expression of this class C AmpC β-lactamase (Table S3). Plasmid-associated AmpC genes associated with resistance against FOX were identified on four ESBL-E (*Citrobacter* sp. AGR7353, *bla*_CMY-40_; *Citrobacter* sp. AGR7360, *bla*_CMY-93_; *Citrobacter* sp. AGR7361, *bla*_CMY-152_; *Enterobacter* sp. AGR6720, *bla*_ACT-55_). Isolates with multiple pAmpC, *bla*_CTX-M_, and *bla*_OXA-181_ (low-level carbapenemase) beta-lactamase genes displayed resistance to 3GC but tested negative in ESBL-double disc confirmatory assays, likely due to the masking of the ESBL activity by the combined hydrolytic effects of the pAmpC and *bla*_OXA-181_ genes not inhibited by clavulanate. The five *Klebsiella* and *Enterobacter* isolates possessed the *oqx*AB genes, encoding an efflux pump, but they were absent in the three *Citrobacter* isolates. Although no quinolone resistance was associated with point mutations in *gyr*A, *gyr*B, or *par*C, moderate ciprofloxacin resistance phenotypes and reduced susceptibility were associated with the presence of *qnr*B and/or *qnr*S genes identified in seven of the non-*E*. *coli* ESBL-E. However, no synergistic effects were observed with the combined *oqx*AB and *qnr*B/S genotypes in *Klebsiella*.

## DISCUSSION

Environmental surveillance of AMR bacteria, such as 3GC-E, is a key component of establishing potential infectious disease transmission pathways between One Health interfaces (8, 73). Understanding the prevalence of clinically relevant bacteria and target ARGs can be established through sampling at the individual human case, animal, community, or farm level, but in many situations, waterbodies, including freshwater and sediment stores, are the final receiving environment for human or animal waste products containing AMR bacteria (74–76). This study made use of freshwater samples obtained from around New Zealand as part of a repeated cross-sectional study to measure *E. coli* concentrations. Selection of urban sites was undertaken to avoid locations impacted directly by point source discharges of processed human wastewater. The multiple land uses surveyed in this study enables the investigation of alternative land uses such as livestock and their role in potential transmission of AMR bacteria.

3GC-E, and in particular ESBL-Ec, are a suitable One Health target to explore putative transmission pathways between humans, animals, and the environment (6–8). Although ESBL-Ec have been identified in New Zealand from clinical samples, especially UTI cases (12, 77), and from previous studies examining freshwater (25–27, 40), their identification from livestock is rare (15, 17, 78). Compared to overseas data, New Zealand is a relatively high user of antibiotics in humans, but overall, a low user of antibiotics for livestock production, with antibiotics prohibited for prophylactic application (18, 19). For example, for the 2024 year, sales of critically important antibiotics in animal production were 65% lower than the average for the previous 5 years; quantities of 3GC and 4GC sold decreased by 18% to 75 kg and accounted for 0.2% of the total antibiotic sales and 5% of critical antibiotic sales (20). Thus, alongside the pasture-based management strategies for dairy, beef, and lamb production, the judicious use of antibiotics in New Zealand is likely to contribute to the low levels of AMR associated with the agriculture sector.

Previous work, including a subset of Phase 3 freshwater samples, determined that higher *E. coli* diversity, measured by phylotype analysis, reflected contributions from a broader range of fecal inputs and was linked to increased *E. coli* concentrations (Log_10_MPN), reinforcing the potential link of land use and source diversity on microbial water quality (30). In the present study, which examined additional Phase 3 samples along with Phase 1 samples, no statistical difference in *E. coli* concentrations was observed between freshwater samples from sites dominated by urban or dairy land use, where *E. coli* levels were generally highest. This finding is notable because, despite similar *E. coli* concentrations, factors such as local antimicrobial use may influence whether a freshwater sample contains 3GC-E. The scarcity of 3GC-E isolates from dairy, avian, sheep and beef, mixed, and low-impact sites supported their broader classification as non-urban land use. Compared with these non-urban sites, ESBL-Ec were substantially more prevalent in freshwater samples from urban-dominated sites, where high *E. coli* concentrations were also recorded. While the binary classification of “urban” and “non-urban” land-use facilitates interpretation, it necessarily masks ecological heterogeneity within the “non-urban” category. Differences between, for example, agricultural and less-impacted sites may influence microbial composition and resistance patterns and could not be resolved within the current analytical framework. Additionally, while ESBL-Ec encompassing a diverse range of isolates were isolated consistently over the course of the study from some urban land-use sites (e.g., U3 and U5), ESBL-Ec were absent from other urban sites (e.g., U7 and U8). In the absence of known point sources, the basis for these site-specific ESBL-Ec prevalences is unknown but may relate to failing wastewater infrastructure and the seepage of human waste into stormwater pipes draining to local waterways (79). For many of the freshwater samples where 3GC-E were isolated, MST data and identification of human as the dominant fecal source was concordant with land-use data, where 3GC-E were more commonly associated with urban land use. This potential link, together with the low prevalence of ESBL-Ec in non-urban areas such as livestock sites, is consistent with national patterns of antimicrobial use in New Zealand. Several model estimates, such as the association of 3GC-E and land use, were associated with wide confidence intervals, indicating uncertainty in the magnitude of effects. Accordingly, interpretations focus on effect direction and consistency rather than precise effect size estimation. In the same way, while the findings are broadly consistent with human-derived inputs contributing to 3GC-E occurrence in freshwater, the observational nature of this study and the constraints of MST and SNP-based approaches mean that definitive transmission pathways cannot be established.

The prevalence of ARGs associated with resistance to aminoglycosides, tetracyclines, fluoroquinolones, and 3GC among the freshwater ESBL-Ec was generally similar to data from human ESBL-Ec. However, despite the presence of shared STs between the three collections, most STs (29/55, 52.7%), including eight STs from freshwater, were singletons represented by only one isolate, suggesting the broad host-range of Class A *bla*_CTX-M_ ESBL genes. Indeed, ARGs associated with the broader ESBL/pAmpC-resistance phenotype were distributed in diverse freshwater isolates across the *E. coli* phylogeny. In total, 3GC-Ec representing seven *E. coli* phylotypes (A, B1, B2, C, D, E, and F) and 55 STs were identified, suggesting the widespread ARG transfer in intestinal and extraintestinal isolates. ExPEC phylotype B2 was the most common phylotype and included ST131, widely recognized as a globally circulating ESBL-type associated with significant human disease (80–82). ST131 was also the most common ST from the two human ESBL-Ec data sets and has been previously isolated from freshwater in New Zealand (40), including one recent study where it was the most common ST among isolates obtained from effluent, sediment, stormwater, and freshwater samples (25). Unlike overseas, where ESBL-Ec ST131 has been commonly isolated from livestock, including cattle (83–86), ST131 has not yet been isolated from dairy farm studies in New Zealand. Moreover, few direct epidemiological links exist in New Zealand connecting ESBL-Ec from the human and animal One Health compartments. Interestingly, however, one freshwater isolate, AGR6711, containing a chromosomally encoded *bla*_CTX-M-15_ gene as a singleton ARG, appeared to have very similar strain characteristics (ST4553, O83:H42, phylotype F, *bla*_CTX-M-15_ singleton ARG) and be potentially linked to an isolate obtained from dairy effluent in a previous New Zealand study (15). Although obtained from a site where urban was the dominant land use, MST methods demonstrated the presence of both putative human and ruminant fecal sources in the freshwater sample. A limitation of this study is the temporal mismatch between freshwater isolates (2020–2023) and human clinical isolate data sets (2019–2021). As isolates were not collected contemporaneously, phylogenetic proximity cannot be interpreted as evidence of recent shared transmission events or direct epidemiological linkage. Instead, observed genetic similarity may reflect the broader temporal persistence and dissemination of successful ESBL-producing lineages. However, ST4553 harboring *bla*_CTX-M-15_ as singleton ARGs were also identified from the ESR (*n* = 2) and MidCentral (*n* = 1) clinical isolate data sets, and less contemporaneously also from dog feces (*n* = 1) and stormwater (*n* = 3) in New Zealand (26, 77), suggesting common lineages associated with shared or overlapping reservoirs. Although the sparsity of ESBL-Ec isolated from livestock in New Zealand provides strong evidence for parallel circulation across human and environmental compartments, further studies are required to elucidate the dominant transmission pathways.

Enrichment cultures were plated on mCCDA supplemented with cefoperazone as the primary selective agar for 3GC-E. Although overgrowth of ESBL-Ec on mCCDA is a well-recognized confounding factor when isolating *Campylobacter* from poultry or environmental samples (40–42), in this context, it proved advantageous for 3GC-E selection. Despite the benefits of selective culture using mCCDA, Colilert enrichment conditions are likely to favor the growth of particular taxa (e.g., Enterobacteriaceae) and resistance phenotypes, potentially influencing observed community composition and resistance profiles, particularly in low-biomass environments where target organisms are rare or near detection limits. This may contribute to apparent differences in 3GC-E prevalence between urban and non-urban sites; however, the consistent application of methods across sites supports the comparability of observed patterns. Furthermore, unlike media, such as ESBL-CHROMagar or MacConkey agar containing 3GC, which require interpretation of colony color to identify putative ESBL-Ec, growth on mCCDA produces large, pale cream colonies. This simplified the selection of colonies for subculture, eliminating the need for colony-color-based assumptions. Interestingly, many 3GC-Ec isolates recovered from mCCDA and subcultured on ECC-CHROMagar and MacConkey agar displayed atypical non-lactose- and non-β-glucuronidase-positive fermentation profiles, suggesting that they may have been overlooked on standard media (87, 88).

WGS data indicated the presence of two freshwater ESBL-Ec (ST410 and ST617) harboring the plasmid-encoded *bla*_NDM-5_*ble*_MBL_ twin gene cassette associated with carbapenemase resistance. Both ESBL-Ec were isolated from the same urban site during October 2022 and May 2023, but none of the human ESBL-Ec isolates included in this study harbored *bla*_NDM-5_. Carbapenemase-resistant ESBL-Ec, including ST410 and ST617, have been isolated from humans and animals in overseas studies and are often associated with a MDR phenotype (89–91). Clinical carbapenemase-producing Enterobacterales (CPE) were first isolated from humans in New Zealand in 2009 (92), with 78 distinct CPE isolated from 53 patients in 2022 (92, 93). CPE have also been isolated from raw sewage, effluent, oxidation pond water, and sediment from a wastewater treatment plant in New Zealand (28), but this study is the first to isolate clinically relevant epidemic CPE from freshwater and indicates potential transmission pathways from humans to the environment through damaged or inefficient wastewater infrastructure.

There was a diverse array of virulence factors (*n* = 185) identified from the 501 ESBL-Ec included in this study, with many, such as *sit*A, *iro*N, *iuc*A, *iut*A, and *pic,* from ExPEC phylotype B2 associated with iron scavenging and sequestration (94, 95). Shiga toxin-producing *E. coli* (STEC, carriage of *stx* genes) and Enteropathogenic *E. coli* (EPEC, carriage of *eae* gene) pathotypes harboring ESBL genes were not identified. However, two freshwater ESBL-Ec (AGR6717, ST131 and AGR7381, ST8131) were isolated possessing *agg*ABCDR genes responsible for enteroaggregative adherence (EA) from the enteroaggregative *E. coli* (EAEC) pathotype (96, 97). pAA is a well-characterized plasmid containing several genes encoding aggregative adherence fimbriae (AAF) responsible for the stacked brick EA phenotype of EAEC on cultured epithelial cells (98, 99). Horizontal gene transfer of a pAA-like plasmid, alongside acquisition of *stx2a*, drove the emergence and virulence of the STEC/EAEC O104 hybrid pathotype responsible for the 2011 European outbreak (99, 100). More recently, mobilization of similar plasmids into ESBL-Ec ST131 was reported in bacteraemia and UTI isolates (101, 102), with loss of AAF-encoding genes associated with reduced adherence to colonic epithelial cells (101). ESBL-Ec containing *agg* genes were also identified in human isolates from the ESR data (*n* = 1) and the MidCentral data (*n* = 3), pointing to potential environmental contamination with human ESBL-Ec/EAEC hybrid pathotypes (103). These findings demonstrate the movement of the pAA plasmid and AAF-encoding genes across distinct One Health interfaces and among different *E. coli* pathotypes, highlighting EAEC-specific virulence traits in human ESBL-Ec that may enhance intestinal colonization.

The use of long-read sequencing (ONT) was able to produce complete, contiguous genome assemblies, overcoming the limitations of traditional short-read methods. For example, long-read assemblies were able to discern that some ESBL-Ec were devoid of plasmids and that all ARGs present in these isolates were chromosomal. Observations such as these are critical for supporting genomic epidemiology, improving source attribution analyses, and assessing overall AMR risk. The assembly of whole chromosomes and complete plasmids with high accuracy also provided an indication of phenotype and genotype associations; ciprofloxacin resistance-associated ASTs were able to be classified according to PMQR ARGs or stepwise mutations in QRDRs of *gyrA* and/or *par*C (104, 105).

The use of mCCDA agar also supported the isolation and identification of non-*E*. *coli* ESBL-E, including *Citrobacter*, *Enterobacter,* and *Klebsiella* sp., from freshwater samples, often with an MDR phenotype. However, further analysis to confirm the ESBL phenotype on CPD and CTX of two *Klebsiella* isolates with *bla*_LAP-2_ and *bla*_LEN-17_, respectively, suggested limited hydrolytic activity of 3GC. While ESBL-Ec are the most clinically relevant AMR bacteria, surveillance of non-*E*. *coli* ESBL-E is important to track horizontal gene transfer, assess the relative risk of treatment resistance (e.g., ESBL or pAmpC phenotypes), and monitor both ESBL-Ec-associated ARGs, such as the CTX-M family, and novel resistance genes absent in 3GC-Ec. Of the eight freshwater samples from which non-*E*. *coli* ESBL-E were isolated, seven originated from sites with predominantly urban land use. Interestingly, MST data indicated mixed putative sources of fecal contamination, including human, ruminant, and avian, for six of the water samples, suggesting diverse fecal origins.

Although there was a broad coverage of freshwater samples taken from the main urban and agricultural regions of New Zealand, a limitation of this study was that some regions were more represented than others. Furthermore, no seasonal effects of 3GC-E prevalence were deduced as sampling did not occur throughout a continuous 12-month period. However, independent of sample site, there was a clear association between 3GC-Ec isolation and increased freshwater *E. coli* concentration. Previous data demonstrated the significance of increased rainfall and increased flow on *E. coli* concentrations (30). While the highly dynamic nature of freshwater environments and associated *E. coli* concentrations are heavily influenced by land use, weather events, and water flow rate also impact contaminant levels (106). 3GC-E were more commonly associated with urban areas and with increased rainfall over the preceding 72 h. Given the central role of rainfall in generating overland flow, differences in rainfall-driven transport processes between non-urban catchments and built-up environments, where soil saturation and impervious surfaces respectively dominate runoff generation and transport, alongside potentially higher antimicrobial use in urban settings, may collectively contribute to variation in 3GC prevalence in freshwater, although these relationships remain unquantified.

There are almost 4.7 million cows within 10,500 dairy herds in New Zealand (107); however, evidence from this study, alongside previous on-farm sampling investigations, suggests that at any given time, ESBL-E may colonize only a small number of animals on a farm or occur as an environmental contaminant on a few farms. These observations suggest that if implemented across the livestock production sector elsewhere, antimicrobial stewardship and prudent antimicrobial use can reduce the emergence of AMR bacteria and the spread of ARGs. Thus, combining freshwater sampling and fecal source testing with spatial land-use mapping provides a more holistic One Health approach to establishing potential AMR source attribution and offers a practical alternative to extensive animal and farm sampling in settings where AMR prevalence is low (108). However, when considered alongside the increasing frequency of heavy rainfall events driven by climate change and aging wastewater and stormwater infrastructure, these findings support the hypothesis that there is a significant risk of AMR bacteria transfer from humans to the environment, with this risk varying considerably among urban sites (109, 110). It would be valuable to conduct a parallel overseas study encompassing livestock production areas with higher use of 3GC and 4GC for animal health and welfare. This would enable a comparative assessment of land-use impacts and fecal source tracking of ESBL-E isolated from freshwater, as well as their associated ARGs. Understanding how ARGs are mobilized and maintained within environmental bacteria is therefore critical. In addition, the use of long-read sequencing of freshwater ESBL-E provides crucial insights into the genomic context, revealing whether ARGs and clinically significant virulence factors are located on the chromosome or plasmids, identifying plasmid types, and characterizing nearby mobile genetic elements. Such information clarifies how genes may move through horizontal gene transfer, their potential for mobility, and the co-location of other genes that could be co-selected (e.g., within MDR plasmids). From a One Health perspective, understanding these genomic linkages between environmental, agricultural, and clinical reservoirs in parallel with robust antimicrobial use data strengthens risk assessment and informs surveillance strategies aimed at mitigating AMR transmission across human, animal, and environmental interfaces.

In conclusion, this study demonstrated that the isolation of 3GC-Ec was strongly associated with higher *E. coli* concentrations in freshwater samples. However, while generic *E. coli* concentrations were similarly elevated in both urban and dairy-dominated catchments, the prevalence of 3GC-Ec was markedly higher in urban freshwater, suggesting that high fecal loadings alone are not the cause of AMR occurrence. Instead, one hypothesis is that urban-specific factors, such as higher antimicrobial use and aging or inadequate wastewater and stormwater infrastructure leading to unintended discharges into waterways, are likely drivers. WGS of 3GC-Ec isolates also revealed epidemiological links to other One Health compartments, particularly human-associated ESBL-Ec, at both the ARG and virulence gene levels. These findings underscore the likely infectious disease risks posed by the environmental dissemination of clinically relevant ESBL-Ec lineages and highlight the urgent need for remediation and upgrading of urban infrastructure to reduce the potential for AMR transmission from urban areas into freshwater systems.

## Data Availability

Whole genome sequencing short and long read data for the freshwater 3GC-resistant Enterobacterales are deposited in GenBank under NCBI BioProject number PRJNA1367133 (SAMN53331561-SAMN53331603). Short read data for ESR and MidCentral ESBL-Ec are deposited in GenBank under NCBI project numbers PRJNA1077717 and PRJNA1032159, respectively.

## SUPPLEMENTAL MATERIAL

Supplemental figures aem.00242-26-s0001.docx Fig. S1 to S7.

Supplemental tables aem.00242-26-s0002.xlsx Tables S1 to S9.

ASM does not own the copyrights to Supplemental Material that may be linked to, or accessed through, an article. The authors have granted ASM a non-exclusive, world-wide license to publish the Supplemental Material files. Please contact the corresponding author directly for reuse.
