# Supplementary material for: Linking land use to the likely origins of third-generation cephalosporin-resistant Enterobacterales in freshwater
Source: Appl Environ Microbiol. 2026 Jun 26;92(7):e00242-26. doi: 10.1128/aem.00242-26 (PMC13390354; doi:10.1128/aem.00242-26)

**Supplementary Figures.**

Linking land-use to the likely origins of third generation cephalosporin-resistant Enterobacterales in freshwater.

Running title: Land-use links to freshwater 3GC Enterobacterales origins

Adrian L. Cookson^1,2,#^, Rose M. Collis^1^, Meg Devane^3^, Jonathan C. Marshall^4^, Marie Moinet^1,*^, Amanda Gardner^1^, Lynn Rogers^1^, Sara Burgess^2^, Patrick J. Biggs^2,5^, Brent J. Gilpin^3^.

^1^ New Zealand Institute for Bioeconomy Science, AgResearch Group, Hopkirk Research Institute, Massey University, Palmerston North, New Zealand.

^2^ mEpiLab, School of Veterinary Sciences, Massey University, Palmerston North, New Zealand

^3^ New Zealand Institute for Public Health and Forensic Science, Christchurch, New Zealand

^4^ School of Mathematical and Computational Sciences, Massey University, Palmerston North, New Zealand

^5^ School of Food Technology and Natural Sciences, Massey University, Palmerston North, New Zealand

**^#^ Corresponding author:** Adrian Cookson, Email: [adrian.cookson@phfscience.nz](mailto:adrian.cookson@agresearch.co.nz)

***Present address:** New Zealand Institute for Public Health and Forensic Science, Porirua, New Zealand

**Fig. S1.** New Zealand water sampling sites and associated dominant land-use included as part of this study. For Phase 1 water samples were obtained between 13 February and 19 March 2020 from 16 sites around New Zealand. For Phase 3, freshwater samples were taken between October 2022 and July 2023 at 41 sites around New Zealand. Eight sites from Phase 1 (n= 4 dairy, n= 2 sheep and beef, n= 2 urban) were also included in the 41 sites sampled during Phase 3.


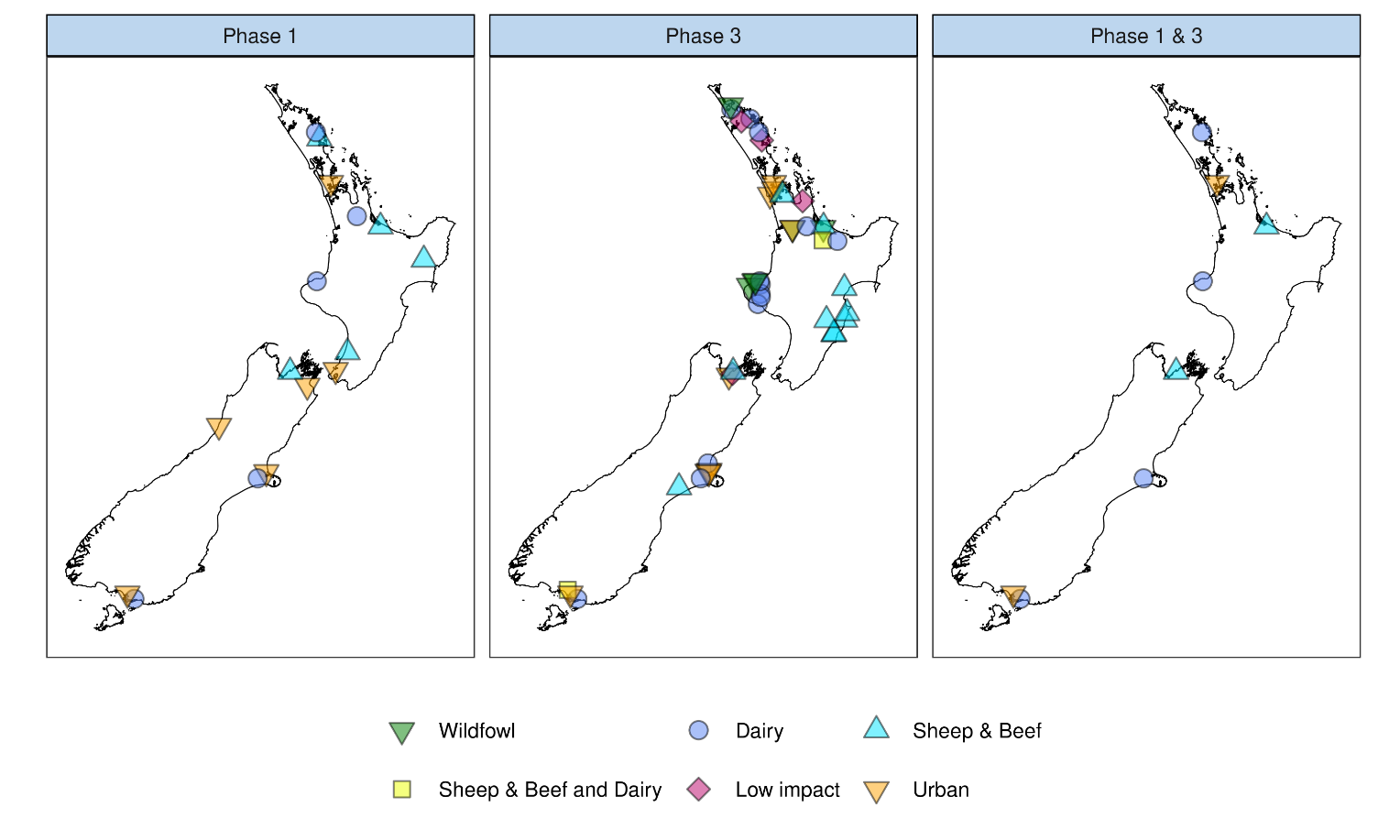


**Fig. S2.** *E. coli* Log_10_ MPN per 100 mL concentrations across sites. Box and whisker plot of log_10_-transformed freshwater *E. coli* (MPN per 100 mL) counts (n=340) across different dominant land-use. Points represent individual measurements from each land-use. The boxes show median values and span lower to upper quartiles, the whiskers show the highest and lowest values within 1.5 times the interquartile range, and dots beyond the whiskers show potential outliers. Pairwise comparisons between Urban and other land-use sites were performed using Wilcoxon rank sum-tests, with significance indicates as: p < 0.05 (*), p < 0.01 (**), p < 0.001 (***), p < 0.0001 (****), ns – not significant.


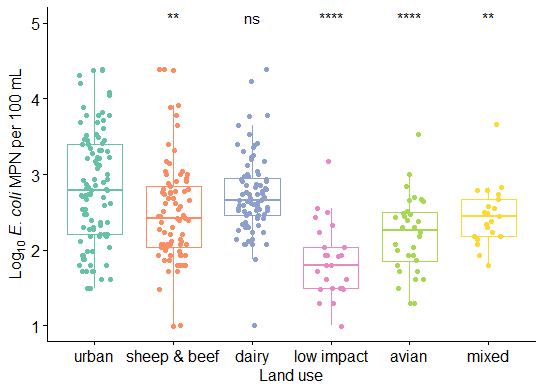


**Fig. S3.** Co-occurrence network of antimicrobial resistance genes (ARGs) detected across freshwater ESBL-*E. coli* isolates. Each node represents an ARG, coloured by antibiotic class (e.g., β-lactams, quinolones, aminoglycosides, macrolides, folate pathway antagonists). Edges indicate significant co-occurrence relationships (Spearman’s ρ > 0.6, p < 0.05) identified using pairwise correlation analysis. Node size is proportional to the gene’s degree (number of connections), reflecting its co-occurrence frequency. The network was visualised in R using the igraph package, with layout determined by the Fruchterman–Reingold algorithm. Highly connected nodes such as *bla*_CTX-M-15_ and *bla*_CTX-M-27_ indicate potential multi-drug resistance linkages.


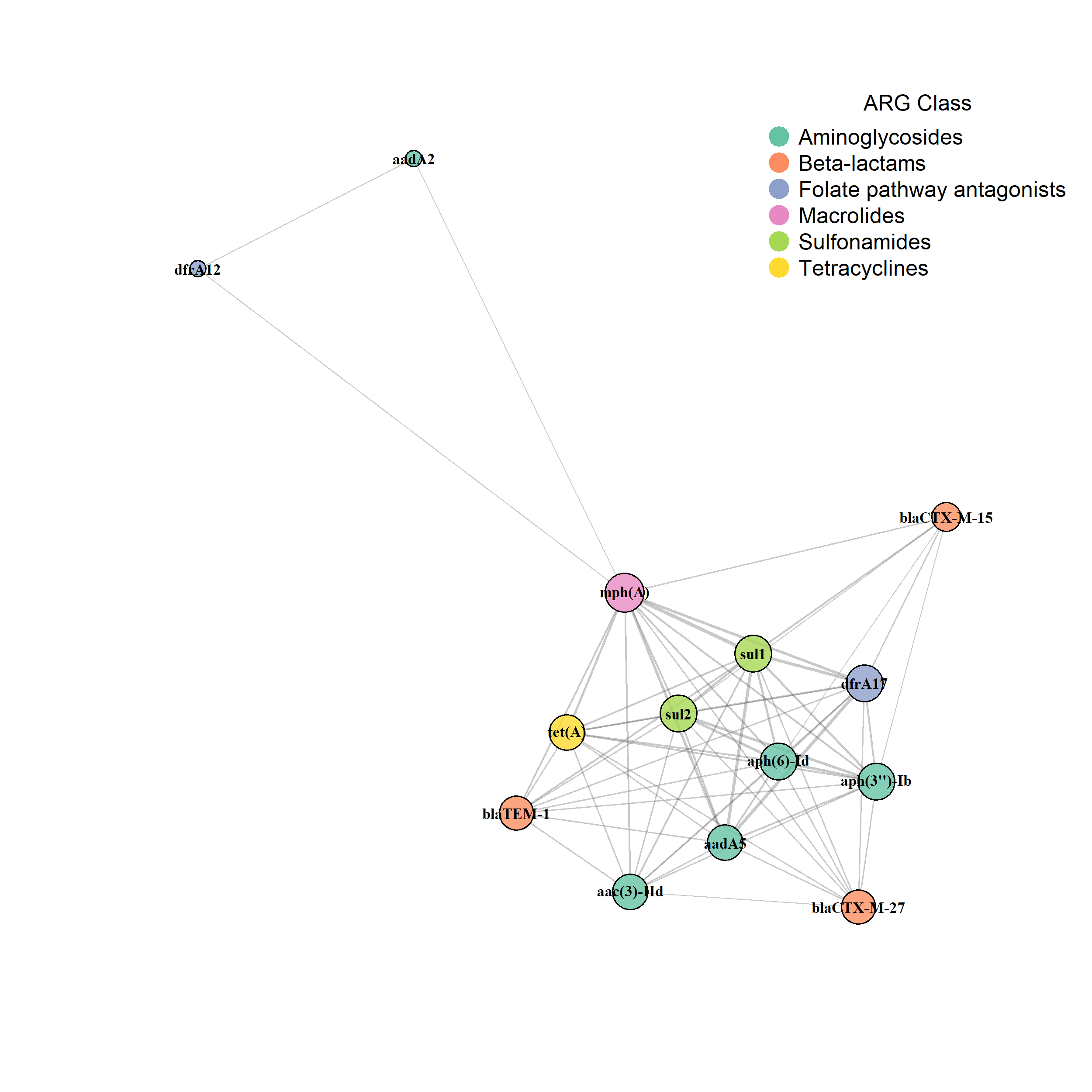


**Fig. S4.** Distribution of ciprofloxacin inhibition zone diameters among ESBL-producing *E. coli* with different quinolone resistance genotypes.
Dot plot showing the overall distribution of ciprofloxacin (CIP) disc diffusion zone diameters (mm) for ESBL-producing *E. coli* isolates. Individual data points are overlaid and differentiated by symbol according to the detected CIP resistance genotype (plasmid-mediated quinolone resistance genes - *qnr*B, *qnr*S, and SNPs in the quinolone resistance determining region - *gyr*A, *par*C variants, or combinations thereof). Dashed horizontal lines indicate the EUCAST clinical breakpoints for sensitive (≥ 26mm), intermediate (22–24 mm) and resistant (≤21 mm) categories. The plot highlights that isolates carrying plasmid-mediated quinolone resistance genes generally exhibit reduced inhibition zones compared with isolates lacking these determinants.


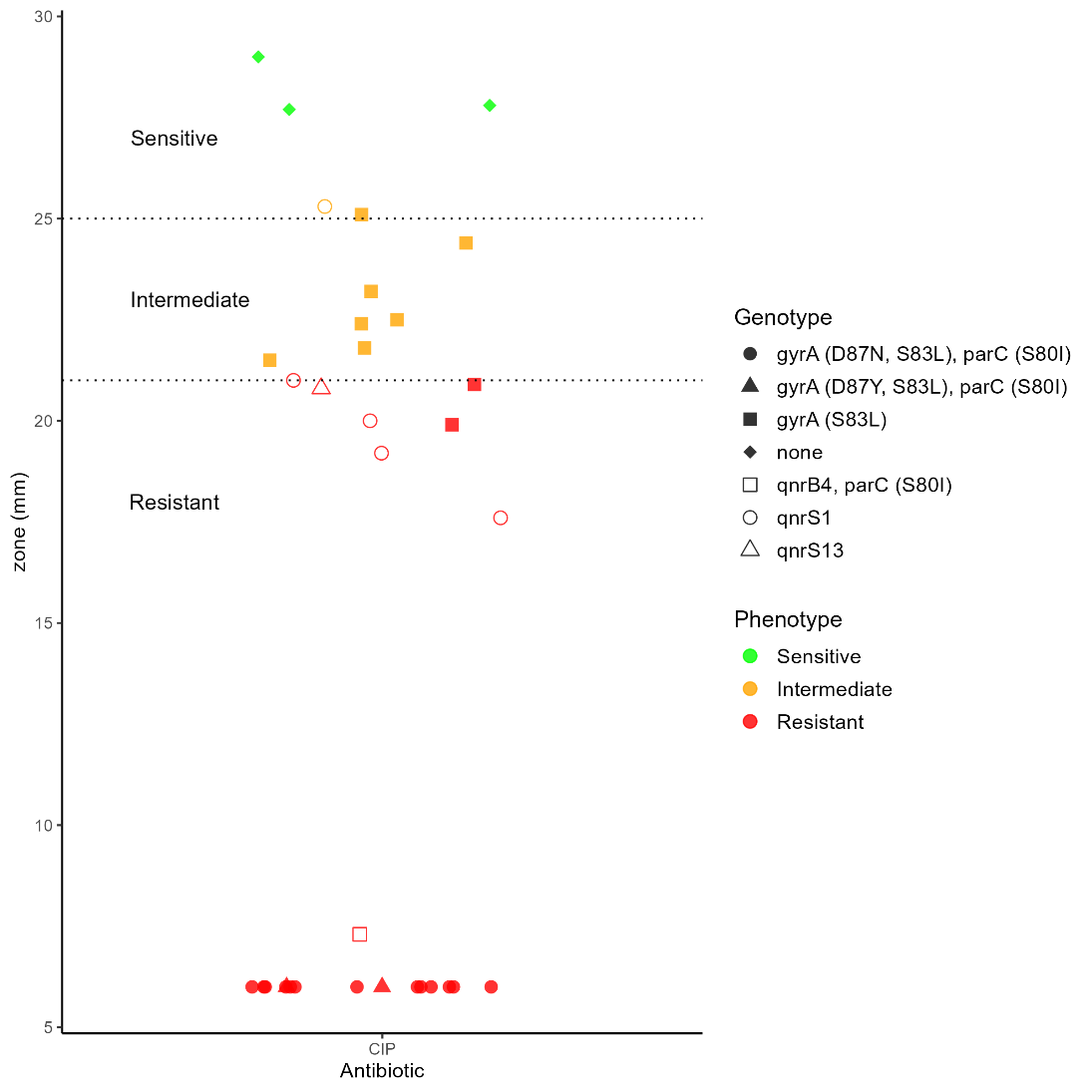


**Fig. S5.** Core genome MLST phylogenetic relationships among third generation cephalosporin-resistant *E. coli* isolate collections from QMRA (n=34), ESR (n=160), and MidCentral (n=367) datasets. Core-genome multilocus sequence typing (cgMLST) analysis was performed using chewBBACA, and the resulting minimum spanning tree allelic profiles were visualised in GrapeTree. Each node represents an isolate, coloured by (a) sequence type (ST) or (b) source. The branch lengths have been collapsed to show isolates falling within ≤ 50 cgMLST allelic differences as a single pie chart node. Branch lengths correspond to relative genetic distances between isolates, representing the number of differing core loci. The branch length is indicated by the scale bar at the base of the tree.

(a)


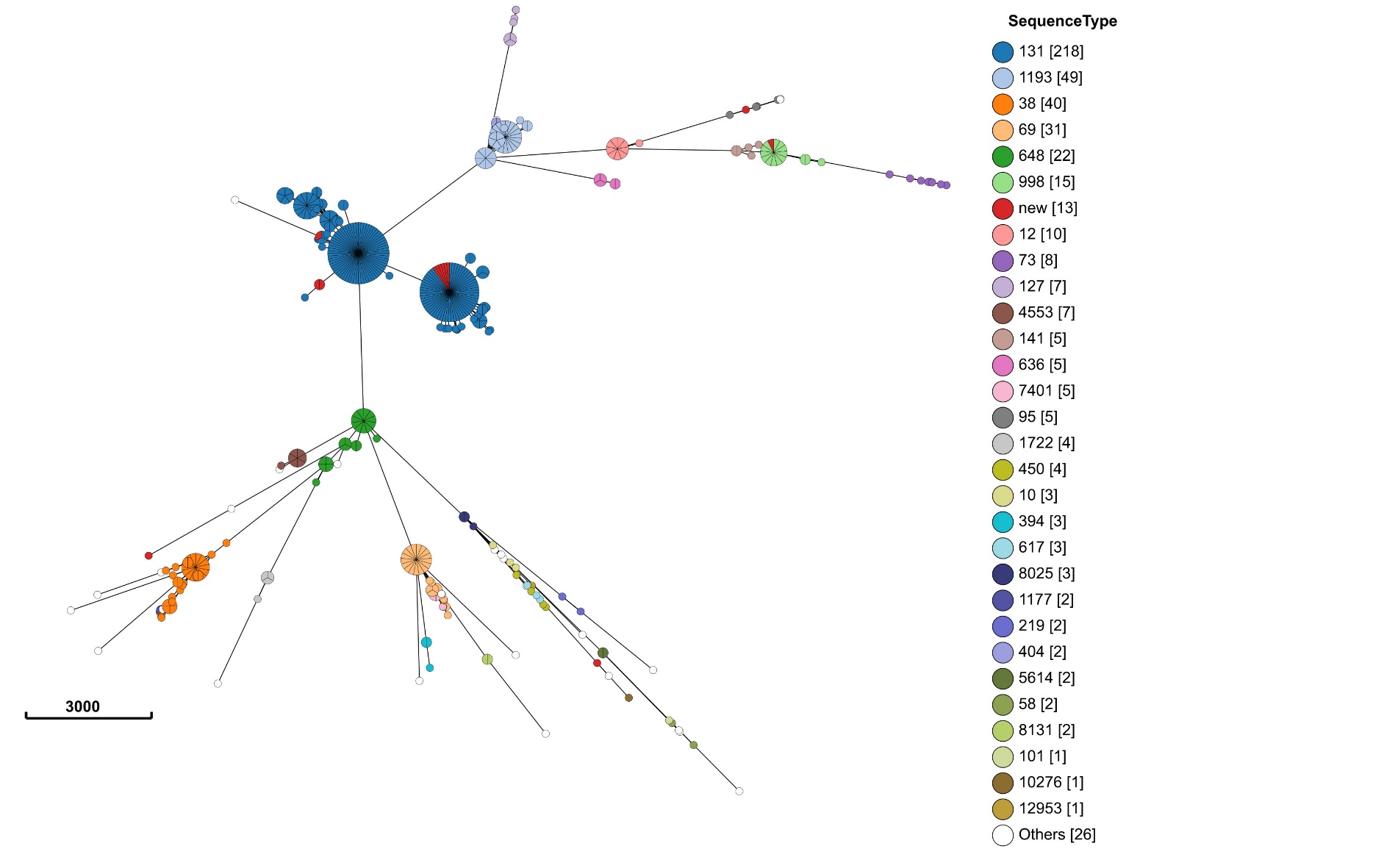


(b)


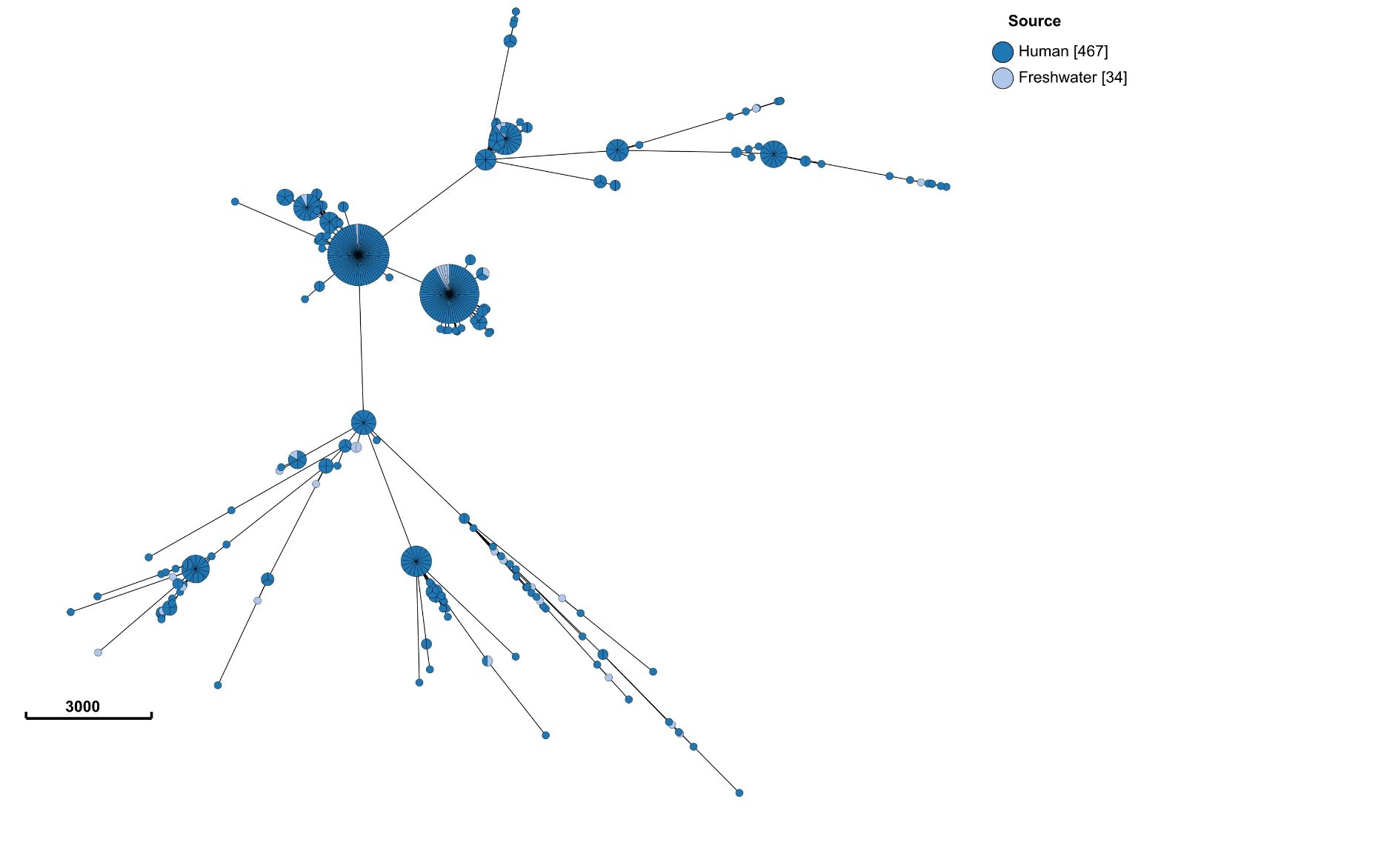


**Fig. S6.** Comparative plasmid analysis of five multi-drug resistant (MDR) plasmids using BRIG (BLAST Ring Image Generator).
The circular map illustrates the structural comparison of five plasmids (Col156, IncFIB, IncFII) from ESBL-*E. coli* ST131 Clade B harbouring an MDR genotype. The innermost ring represents the reference plasmid pAGR6715a from ESBL-*E. coli* AGR6715, while concentric outer rings depict plasmids pAGR7351a from ESBL-E. coli AGR73751, pAGR7362a (AGR7362), pAGR7368a (AGR7368), pAGR7386a (AGR7386) aligned by BLASTn similarity. Colour intensity of the rings reflects sequence identity, with darker shading indicating higher similarity. The map highlights regions of high degreee of conservation of plasmid nucleotide sequence including the antibiotic resistant genes *dfr*A17, *sul*1, *sul*2, *aph*(3’’)-Ib, *aph*(6)-Id, *tet*(A), and *bla*_TEM-1B_.


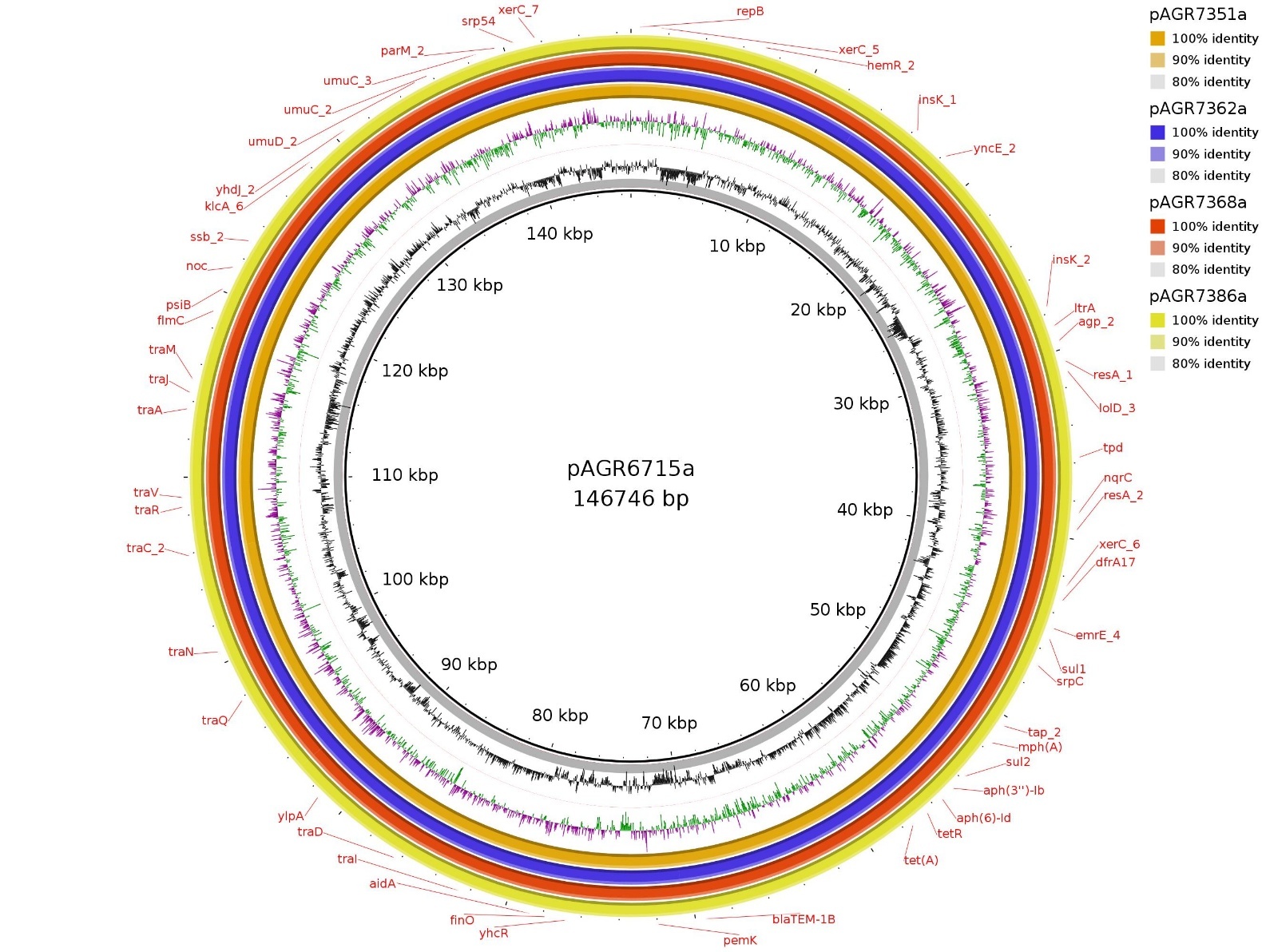


**Fig. S7.** Genetic map of a large multidrug-resistance plasmid (IncFIB, IncFIC) pAGR7389a from ESBL-*E. coli* AGR7389.
The circular plasmid diagram illustrates the location and organisation of antimicrobial resistance genes (ARGs) and virulence-associated genes. ARGs are colour-coded red, while virulence genes are purple. The map demonstrates co-localisation of clinically relevant ARGs and virulence factors, suggesting the plasmid may contribute simultaneously to antimicrobial resistance and pathogenicity.


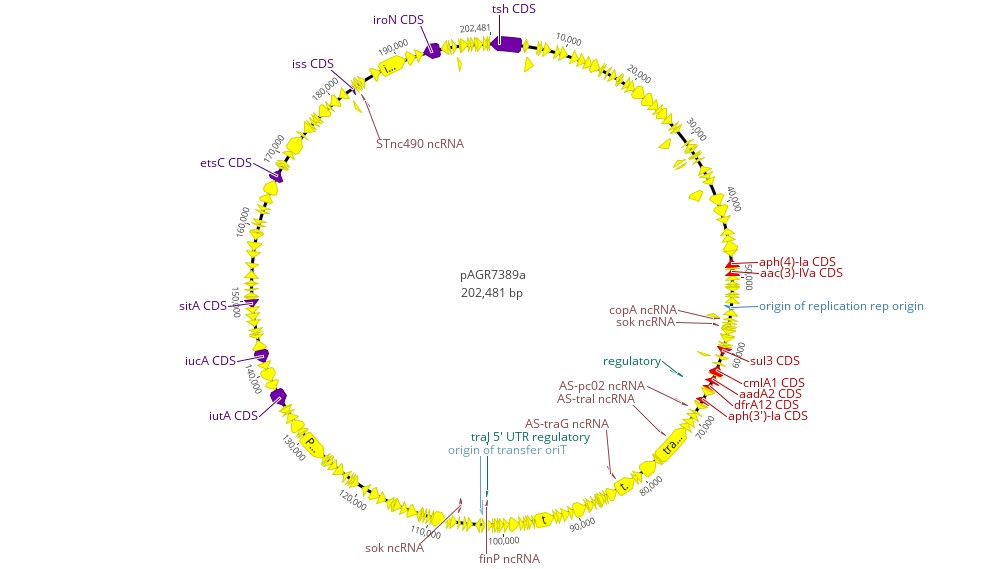

Supplement: Supplemental figures — Fig. S1 to S7. [file aem.00242-26-s0001.docx]
